# Text Psychometrics: Assessing psychological constructs in text using natural language processing

**DOI:** 10.1371/journal.pdig.0001757

**Published:** 2026-11-12

**Authors:** Daniel M. Low, Patrick Mair, Matthew K. Nock, Satrajit S. Ghosh

**Affiliations:** 1 Child Mind Institute, New York, New York, United States of America; 2 Department of Psychology, Harvard University, Cambridge, Massachusetts, United States of America; 3 Department of Psychiatry, Harvard Medical School, Boston, Massachusetts, United States of America; 4 Franciscan Children’s Hospital, Boston, Massachusetts, United States of America; 5 Department of Epidemiology, Harvard T. H. Chan School of Public Health, Boston, Massachusetts, United States of America; 6 McGovern Institute for Brain Research, Massachusetts Institute of Technology, Cambridge, Massachusetts, United States of America; 7 Department of Otolaryngology–Head and Neck Surgery, Harvard Medical School, Boston, Massachusetts, United States of America; Shanghai United Imaging Intelligence Co Ltd, CHINA

## Abstract

Large language models (LLMs) have revolutionized natural language processing (NLP). Yet when used to assess psychological constructs in text, they are generally not evaluated for the types of validity, reliability, and standardization typically expected from traditional questionnaires with rating scales. This study bridges that gap by proposing a framework for evaluating the psychometric properties of NLP-based psychological assessments (including LLMs-based ones), an area of research we refer to as Text Psychometrics. We first review different NLP methods, compare their ability to address key challenges in psychological research such as explainability, and outline ways of evaluating them on many desirable psychometric properties. Our goal is to describe most psychometric properties that may be of interest, especially for psychology and NLP researchers who are less familiar with psychometrics or psychometricians who are less familiar with NLP. We then illustrate the tradeoffs of different NLP methods through two empirical studies. Study 1 classifies thousands of crisis counseling conversations and Reddit posts into different types of mental health issues and introduces a novel method to evaluate text models for content validity—the extent to which a test captures the full range of expressions of a single construct. Study 2 examines prospective criterion validity by estimating how 49 known suicide risk factors predict imminent risk in crisis counseling conversations. We find that mentioning lethal means for suicide, the police, depressed mood, and anxiety in text as well as the texter writing more than the counselor characterize the conversations that end up needing an emergency service intervention for imminent suicide risk, which showcases the interpretability of the method that was used. In sum, NLP studies in psychology often rely on only a few validation metrics; here, we demonstrate the need for broader psychometric evaluation and, as a step towards Text Psychometrics, provide practical examples and future directions that can help researchers evaluate these models.

## Author summary

Psychological concepts—such as depression, anxiety, or suicide risk— are increasingly being measured by analyzing natural language from clinical transcripts and social media using artificial intelligence. While traditional questionnaires undergo rigorous testing to ensure they are valid, reliable, and fair, artificial intelligence models evaluating text are rarely subjected to the same level of scrutiny. Often, these models are only evaluated on whether they can accurately predict a human-provided label on the same text data, ignoring other crucial properties like whether the model captures the full range of a symptom or performs fairly across different demographics. In our paper, we promote evaluating the validity of computational psychological assessments from text data in a broader way than what is typical, an endeavor we refer to as “Text Psychometrics.” We provide practical examples which can help researchers when evaluating these models. Through two empirical studies analyzing crisis counseling conversations and social media posts, we demonstrate how to test these models for their ability to capture comprehensive symptoms and predict future risks. We find that while some models predict human labels well, they may still miss obvious, core expressions of the symptom. Ultimately, our framework guides researchers in selecting and validating text-based tools to ensure they are safe, effective, and scientifically sound.

## Introduction

Text data is ubiquitous: it is present in social media, direct messages, clinical interview transcripts, survey responses, electronic health records, and scientific documents. There are thousands of distinct well-studied constructs psychologists and social scientists might wish to quantify in text including emotions, moods, symptoms, attitudes, states, traits, and abilities, often through different dimensions such as valence, arousal, frequency, and severity (i.e., dimensions are a measurable axis a construct varies along) [1]. The goals of these assessments could be to screen for a health outcome, summarize a person’s experience, trigger an intervention, or use measurements as variables in subsequent models to further understand a psychological theory [2,3]. This can be used to make individual-level predictions, such as a suicide risk screening [4], or to do large-scale public-health monitoring such as understanding how a pandemic might be affecting different psychiatric populations over time [5]. The traditional approach Psychometrics has taken to measure psychological constructs has heavily relied on rating scales (Fig 1), alongside behavioral tasks, implicit motive assessments, and other types of data. Rating scales ask a rater to convert a subjective observation into a specific value, usually on an ordinal scale. Compared to rating scales, systematically measuring constructs from natural language itself can seem more complex or inexact. However, language has been shown to describe nuances and extremes with more detail and greater ecological validity than rating scales [2]. Natural language processing (NLP) offers many tools to quantify these constructs, and the recent growth in abilities of large language models (LLMs) –a subset of NLP models based on deep neural networks that have billions of parameters– shows promise for identifying constructs more reliably [2,3].

**Fig 1:**
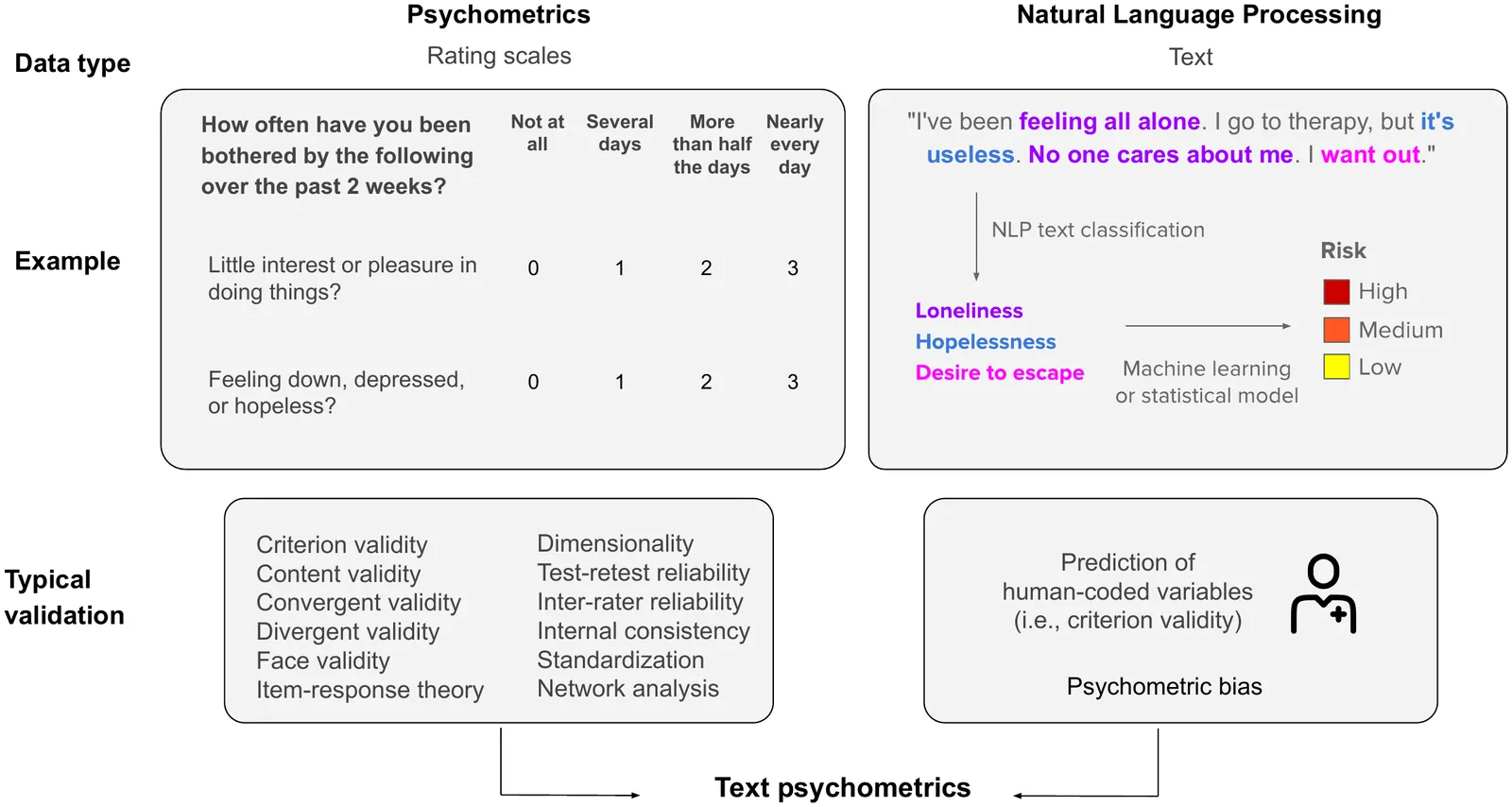
Common types of data and validation in psychometrics and natural language processing. Psychometrics (left) often focuses on using responses to rating scales to assess psychological constructs such as these from the PHQ-2. Natural language processing (NLP; right) can be used to create text models that output whether a text document expressed a given psychological construct. These outputs can be used as is (e.g., useful for flagging high-risk terms) or as independent variables in subsequent machine learning or statistical models (e.g., for risk assessments). While researchers tend to validate rating scale assessments on many psychometric properties using rich modeling choices, NLP studies in psychology mainly report the prediction of human-coded variables. This validation gap implies NLP suffers from a “psychometric bias,” which Text Psychometrics could bridge. Definitions and examples of types of validity, reliability, and models are provided in the section “Desirable psychometric properties of psychological assessments and how to evaluate these properties in text”. Icon has an open source MIT license.

Comparing the utility of natural language and rating scales requires evaluating multiple desirable psychometric properties (e.g., comparing the predictive abilities of each), considering the trade-offs in the time it takes to produce scores (e.g., one-minute journaling entries may capture many more diverse symptoms across individuals than a one-minute screening tool), and assessing how well a researcher can measure a specific construct’s presence and severity. For instance, a person may not mention a specific symptom in an open-text response, but may nevertheless be experiencing it to a certain degree. If it is critical that a specific construct is explicitly captured, or if the individual has difficulty articulating their emotions (e.g., due to severe depression and/or alexithymia), it may be better assessed through a quick ordinal rating scale –though recent evidence indicates language models can actually outperform rating scales even for individuals with high alexithymia [6]. Conversely, natural language is the gold standard in scenarios where self-reported ratings are inadequate; for example, implicit motives or subconscious associations cannot be captured through rating scales, but they often are expressed in natural language or behavioral tasks. Furthermore, open-ended language provides crucial context*—*differentiation, for instance, normative sadness at a funeral compared to clinical depression—which rating scales often strip away.

Historically, the different types of validity, reliability, and standardization typically expected from questionnaires using rating scales [7] are not normally carried out when evaluating NLP models for psychological measurement. Researchers would never publish scales without assessing these psychometric properties, but they do so for NLP and other behavioral or biological markers, which are also susceptible to poor psychometrics, a double standard that has been called the “psychometric bias” [8].

### Validation in natural language processing and machine learning: Correlation does not imply prediction

NLP studies tend to validate models using a single –albeit important– approach: comparing text model outputs to human-coded labels or annotations taken as “ground truth” ([9–11]; see criterion validity in the section “Desirable psychometric properties of psychological assessments and how to evaluate these properties in text”). For instance, humans may code or label sentences from Reddit for different emotions, and then a machine learning model can learn to predict human-coded emotions from patterns in language using a variety of methods [12]. Here, “training” (or “fitting”) a model means adjusting its internal parameters on a set of labeled examples so that its outputs come to match the labels; “inference” is then applying the fitted model to new text. This same approach— predicting human-coded labels— is also the main form of validation used in NLP benchmark leaderboards. Leaderboards allow users to compare and rank models evaluated on many datasets and tasks. For instance, the Massive Text Embedding Benchmark covers 8 tasks (e.g., text classification, summarization, semantic similarity), 58 datasets, and 112 languages [13].

In NLP and machine learning, predictive ability is evaluated on new data than what the model was trained on in order to test generalizability. However, psychology often uses a different and controversial definition of “prediction” [14–16]. Correlating two variables or fitting a linear regression to the data and using the model to provide a predicted value of the dependent variable for a specific individual is not necessarily prediction for machine learning. In those cases, the model “observes” all the data to provide an estimate (i.e., within-sample prediction; see Fig 2). Prediction for machine learning is defined as fitting the model with certain data (i.e., allowing the model to learn the association between dependent variables and independent variables) and then predicting (i.e., “guessing”) the value of the dependent variable for new data the model did not use for any aspect of model fitting (i.e., test set/s) [14]. This is known as out-of-sample prediction and can be done via cross-validation (i.e., splitting the existing data into a training set the model learns from and a separate test set, held back and never seen during fitting, used only to score the model) or other resampling methods [17]. Association can be thought of as a broader term that can refer to (a) this out-of-sample prediction, (b) estimation of the dependent variable’s value for a specific data point using all data during model fitting (i.e., within-sample prediction or association), or (c) estimation of sample-level or aggregate coefficients (e.g., correlations, within-sample regression coefficients, and other within-sample associations). The latter two forms of association are often referred to as “prediction” in psychology, but these within-sample predictions do not imply out-of-sample prediction capabilities (i.e., generalizability); that is, the estimates one obtains from within-sample predictions may not generalize to other similar samples, and machine learning requires this be tested to claim “prediction.” Therefore, while it is common knowledge that correlation (or more generally, association) does not imply causation, it is less known among psychologists that correlation (or association) does not imply prediction. Moreover, many have stressed that prediction should be chosen over association in many psychology studies and related fields [14–16]. For instance, any clinical application would require a model to make accurate predictions on new individuals that arrive to the clinic; we simply would not know whether the within-sample predictions are accurate on new patients without proper out-of-sample testing (see also Discussion of dataset shift in the section “Desirable natural language processing model characteristics that can address key constraints in psychological research”).

**Fig 2:**
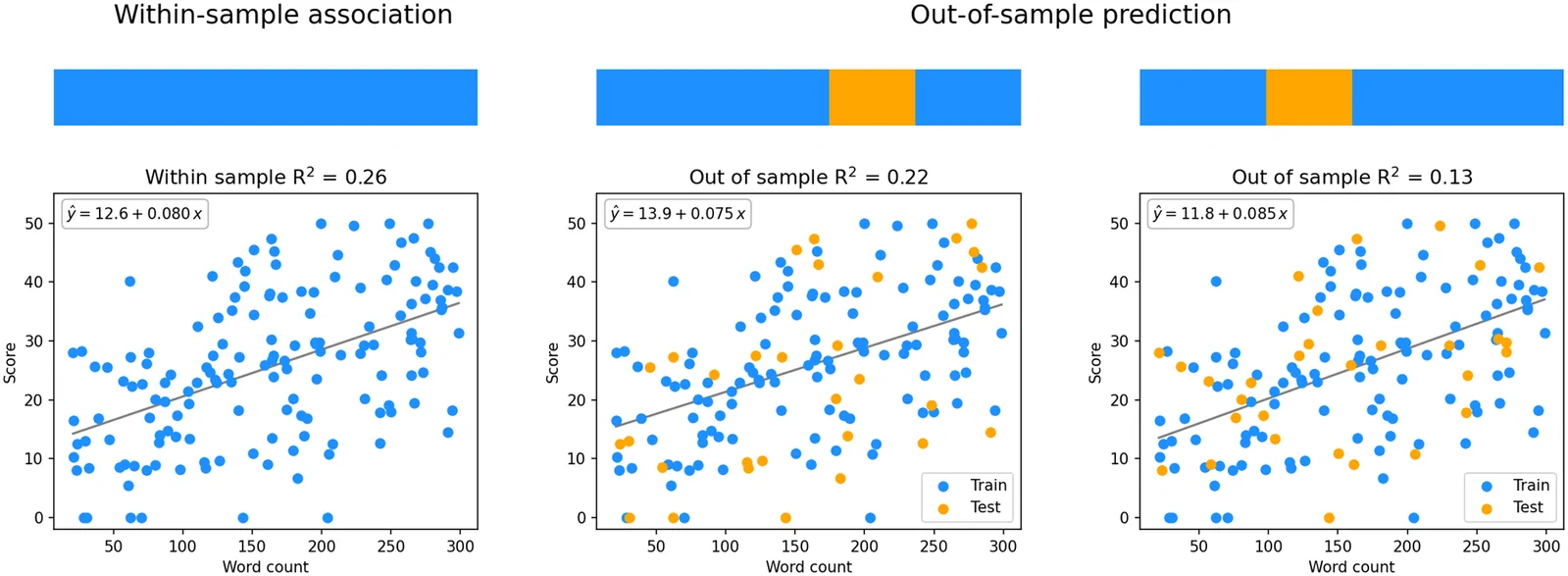
The meaning of "prediction" in psychology and in machine learning. Psychology often uses the term “prediction” for within-sample association (left), where the model is fit and evaluated on the same data. Natural language processing and machine learning reserve “prediction” for evaluation on held-out samples the model did not see during training (out-of-sample prediction of orange data points; center and right), a better measure of generalizability. Because out-of-sample performance depends on which observations are held out, it is typically averaged across multiple splits (cross-validation): here the same model yields R^2^ = 0.22 or 0.13 depending on the test set (orange). Score and word count are illustrative.

Given that the goal of many applications is to try to automate human language tasks, providing a model that generalizes and can predict new samples of human judgments the model has not encountered is understandably the main priority, but should not be the only priority. A single metric is likely not capable of capturing many aspects of validity. We will provide examples of how to evaluate many psychometric properties in the section “Desirable psychometric properties of psychological assessments and how to evaluate these properties in text”. These can be validated through within-sample associations or out-of-sample predictions, with the latter providing a stronger claim of generalizability.

Our study aims to provide a framework for evaluating psychometric properties of NLP models and is structured as follows:

- In the section “Text Psychometrics”, we argue why putting more focus on broader types of validation in NLP-based psychological assessments is important and we review the non-obvious shared history between psychometrics and NLP.
- In the section “Using natural language processing for psychological assessments”, we review different NLP methods and how they can be used to assign a score to a text document (e.g., text classification) for psychological assessments. We also propose key constraints psychology researchers face when using NLP models (e.g., small datasets, the need for explainable and private models) and how to overcome them with different NLP model characteristics. We also introduce an improved method of text classification with embeddings we call “Construct-Text Similarity.”
- In the section “Desirable psychometric properties of psychological assessments and how to evaluate these properties in text”, we provide an overview of how psychometrics has evaluated the validity and reliability of psychological assessments and how these properties can be evaluated in text data.
- In the section “Study 1: assessing multiple types of mental health issues to evaluate concurrent criterion validity and content validity”, we provide a use case and test these methods in detecting a diverse set of mental health issues including suicidal ideation, eating disorders, and physical abuse to compare methods (open-source and proprietary LLMs, lexicons, and embeddings methods) and observe their performance across constructs. We provide a novel way of quantitatively evaluating content validity in text, that is, whether a tool assesses most behaviors or expressions associated with the construct being measured.
- In the section “Study 2: Predicting whether to deploy an emergency service intervention for imminent suicide risk to evaluate prospective criterion validity under class imbalance”, we focus on a methodologically more challenging task, that of predicting which of a set of suicidal individuals is at high imminent risk, which is a scenario with several common constraints in clinical NLP: an imbalanced dataset; and the need for models that are trustworthy and explainable. We predict this using initial parts of the conversation to evaluate prospective criterion validity. We also demonstrate how to inform theory by estimating how predictive each variable is of the dependent variable (i.e., feature importance).
- In the “Discussion” section, we systematically compare NLP methods on a subset of these desirable properties so researchers can decide which NLP method to use, given each method may respond better to the different constraints researchers may have. And we discuss open areas of research for Text Psychometrics.

## Text psychometrics

### Why text psychometrics?

We refer to Text Psychometrics as research into the validation of NLP-based (including LLM-based) psychological assessments derived from human text. This refers to a growing, interdisciplinary research community effort. In this study, we focus on supervised NLP approaches—that is, models trained to predict a known label or score (e.g., a diagnosis, a symptom severity rating). Agreeing upon a specific term may help highlight a critical gap: standard NLP research –while very broad and rich– primarily evaluates out-of-sample prediction of ground-truth labels. While very valuable, this misses the rigorous psychometric requirements (e.g., content validity, reliability, fairness) required for valid psychological assessments.

A related area of research uses NLP to improve the development and validation of rating-scale assessments, such as generating questionnaire items (e.g., [18,19]), which could also fall under Generative Psychometrics (if using LLMs) and under the more established ‘computational psychometrics’ [20], which is much more broad in scope. A second related area of research is assessing the artificial psychological profiles of artificial intelligence (AI) models [21], which has been referred to as AI Psychometrics or LLM Psychometrics [22,23]. We can distinguish this from Text Psychometrics in that the latter measures psychological properties in humans from their natural language or uses NLP to improve rating-scale assessments in humans. AI or generative psychometrics [24] are viable terms but would not be a fitting label for these applications if using many NLP methods (e.g., lexicons, TF-IDF, sentence embeddings) that should not be considered intelligent or generative. Furthermore, AI methods in the distant future may not be called generative.

Independent of how researchers decide to name this type of research –and we are open to other options–, we hope this study will help consolidate efforts to taxonomize and standardize the evaluation of desirable properties of these NLP-based models in a broader sense than what has been done thus far. And there is a growing number of studies trying to improve the psychometric validation of text-based NLP models for psychological assessments [2,9,24–27]. Each study has focused on specific types of models and properties. [11] demonstrated that an NLP model detecting paranoia from text data had good test-retest reliability (see the section “Desirable psychometric properties of psychological assessments and how to evaluate these properties in text” for definitions), it correlated positively with clinical ratings of paranoia and correlated negatively with measures of anxiety and depression. [26] evaluated different types of validity (face, convergent, divergent, content, predictive) of several text embedding models using synthetic natural language data. [27] describe how to assess validity and reliability of NLP model bias such as gender bias. [28] have demonstrated that automated evaluation of topic models using coherence metrics fail to capture human judgments. [29] had annotators rate the degree of hate speech in text documents using an ordinal scale and then used item response theory and deep learning to build a model that outputs a debiased continuous measure of hate speech from text. We contribute to prior work by (1) describing how NLP and psychometrics have a shared history (the section “Bridging natural language processing and psychometrics, two fields with a shared history”), (2) describing traditional and more recent NLP methods and proposing desirable NLP model characteristics (the section “Desirable natural language processing model characteristics that can address key constraints in psychological research”), (3) proposing additional desirable psychometrics properties and methods for evaluating these properties in NLP models (the section “Desirable psychometric properties of psychological assessments and how to evaluate these properties in text”), (4) evaluating a few of these properties across two studies (sections and), and (5) discussing open areas of research and future directions (the section “Discussion”).

### Bridging natural language processing and psychometrics, two fields with a shared history

Human language has played a central role in the historical development of psychometrics. For a brief overview of psychometrics and related methods including item-response theory, network analysis, and causal inference, see section A of S1 Appendix (“Brief history of psychometrics: from classical test theory to networks and causal inference”). In Fig 3 we highlight key developments in psychometrics, NLP, and AI throughout history. Psychometrics has discovered key attributes humans have (e.g., states, traits, attitudes) often by collecting large lists of words associated with psychological traits. Since early in psychometric history, it was clear that just because we have different words for constructs and traits does not mean these words refer to different attributes; therefore, there was a need to discover the underlying independent and general constructs from all possible construct names [30]. Thurstone’s (1934) “The Vectors of Mind” [31] suggested we can discover the key, non-redundant psychological traits by having individuals rate if they would describe someone with adjectives such as “friendly” or “cheerful.” Using factor analysis, he grouped words with similar ratings and was surprised to discover there were only five factors underlying personality. Factor analysis would return how much each word is associated with each factor (i.e., factor loadings). He proposed plotting each adjective as a vector of points in space in five dimensions whereby similar traits would point in the same direction; if they are anticorrelated they would be pointing in opposite directions; and if they are independent, then they would be orthogonal (p. 15). One could measure this using cosine angles from the center of the space. Allport, Odbert, and Cattell between the 1930s and 1950s took this approach and gathered such ratings on thousands of words, an approach that ultimately led to the Big five factors of personality [32], which includes openness to experience, conscientiousness, extraversion, agreeableness, and neuroticism.

**Fig 3:**
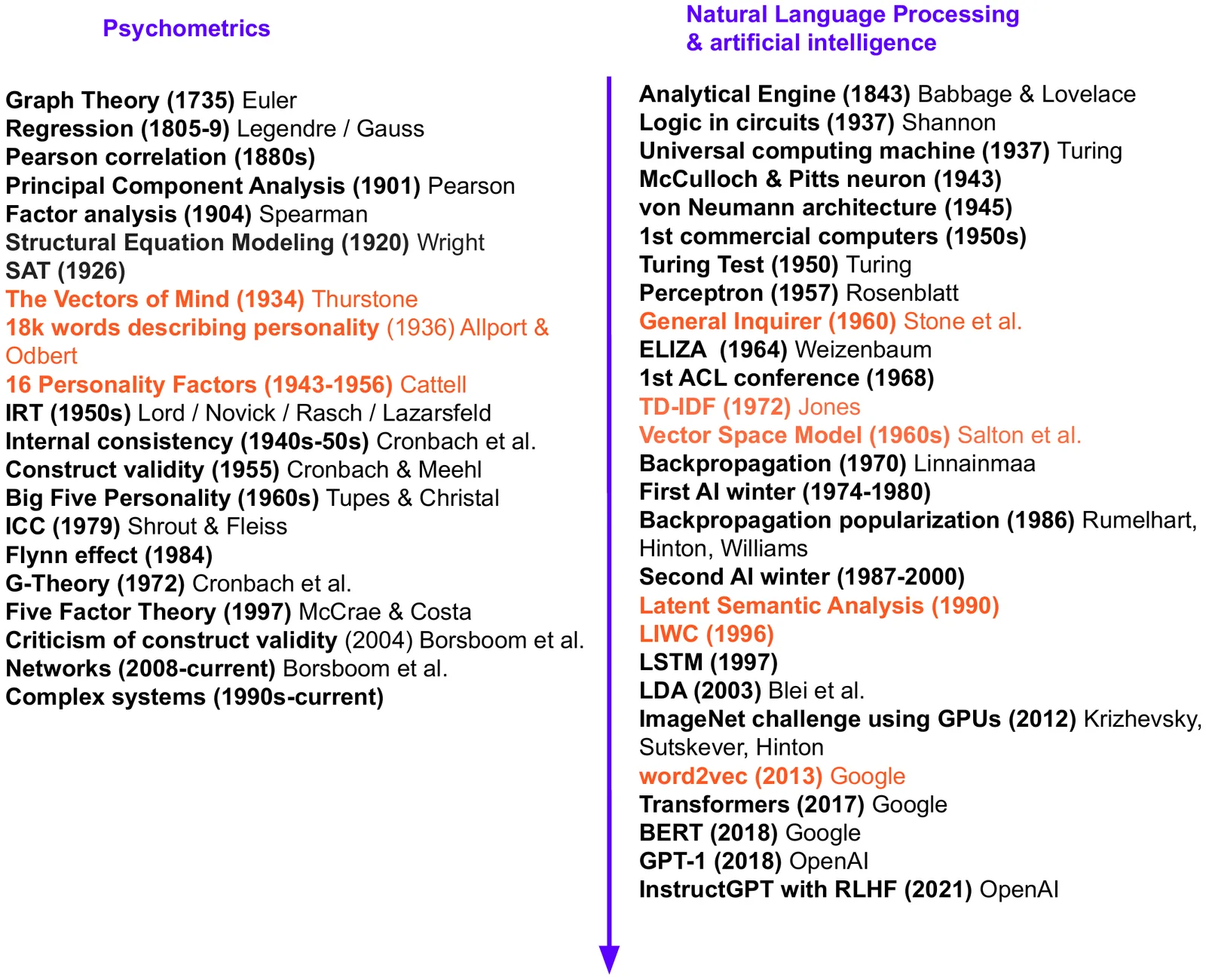
Psychometrics and natural language processing have a shared history. Several innovations in statistics are mentioned as they are the building blocks of psychometrics. Several, non-exhaustive examples of key developments that influenced each other across fields are displayed in orange.

Lexicons (i.e., lists of words and phrases associate with constructs) have also been used to measure psychological constructs in text. The General Inquirer gathered thousands of words related to different constructs, but used the first commercial computers from the 1960s to automatically count the appearance of different words in corpora [33] which later inspired the widely used Linguistic Inquiry and Word Count (LIWC) [34] and many other lexicons [35].

Not only was language key in developing psychometrics, psychometrics has also influenced NLP, although this is relatively unknown. About three decades after Thurstone’s (1934) address, vector space models by Salton and others in the 1960s and 70s took this factor analysis approach [36], (without acknowledging Thurstone as far as we can tell) but vectorized words by counting how many times a word co-occurs with other words (dimensions of the vector) within small windows of text across different corpora [36,37]. This was used to find documents that were similar to a query in information retrieval systems (e.g., finding articles about a topic in libraries).

This approach, along with advances in deep learning and hardware capabilities, contributed to the explosion of vector semantics that came with the creation of the word2vec word embedding algorithm in 2013, which was able to better quantify aspects of the meaning of words in vectors (i.e., ordered lists of numbers that represent positions in a multidimensional space). Improving upon word embeddings led to current state-of-the-art transformer-based embeddings, which can encode aspects of sentence and document meaning. These embeddings from transformer models have a wide variety of uses including information retrieval, paraphrase detection, categorizing text, summarization, and keyword extraction [13]. The origins of vector semantics is often credited to hypotheses in linguistics from the 1950s from Harris (“If A and B have almost identical environments we say that they are synonyms” [38]) and Firth (“You shall know a word by the company it keeps” [39]): vector semantics captured a word’s meaning by counting which words it co-occurs with. However, we find factor analysis as previously described also had a large influence as well. We can add the following quote by Thurstone (1934) to the history of vector semantics: “each trait can be represented as a point on the surface of a ball. If two traits A and B tend to coexist, the two points will be close together on the surface of the ball.” ([31] p. 14).

## Using natural language processing for psychological assessments

### Brief overview of NLP methods for psychological assessments

Conventional psychological assessments tend to provide either binary variables, (e.g., present vs. absent) or continuous scores reflecting severity or frequency of symptoms, which are aggregated for a final score via sum scores, averages, or more complex scoring models (e.g., weighted composites, factor scores from factor analysis, or latent trait estimates from item response theory). How can we assess psychological constructs from text data? NLP offers a diversity of methods. The overall pipeline might involve preprocessing the text data (e.g., tokenization, lemmatization), feature extraction (e.g., turning characters into quantitative information such as counts or embeddings) (In machine learning, ‘feature’ refers to ‘variables’, often independent variables), a specific model (e.g., logistic regression, a specific transformer-based LLM), and output type (e.g., binary, multinomial, ordinal, continuous). Deep learning models including LLMs tend to do any preprocessing within the model. (For more in depth introductions to machine learning, deep learning, and LLMs, see [40–42]).

A recurring question is what the “item” or unit of analysis is in text-based assessment. The input unit that receives a score is the document—a post, conversation, or response— analogous to a test administration, with the construct detector acting as the scorer. Sentences, phrases, and tokens are the finer granularities at which feature extraction operates for particular methods (e.g., construct-text similarity tokenizes into independent clauses). There is thus no fixed “item” analogous to a scale item. A separate, diagnostic notion of item appears later in our content-validity test sets (Section 5.4), where individual prototypical tokens are used to probe coverage.

If the output is categorical (binary or multinomial) then machine learning calls this task ‘classification’. If the output is ordinal or continuous, this is known as a ‘regression’ task in machine learning (not to be confused with the type of model such as linear regression or logistic regression). Classification varies between binary classification (one out of two possible answers is correct, “Does the text mention loneliness?”), multiclass (only one out of several answers is correct, outputs for all options sum to 1; e.g., “Does this text belong to the depression, anxiety, or healthy participant samples that do not overlap?”), and multilabel (more than one answer can be correct, output for each range from 0 to 1; e.g., “Which symptoms does the text mention?”). (For a more formal description of text classification, see section C of S1 Appendix (“Formal description of text classification”).). A regression task (i.e., predicting an ordinal or continuous output) could try to detect severity from text (e.g., total score of the PHQ-9 from a concurrent journal entry), frequency (e.g., how often the person felt sadness in the last two weeks), or the model’s confidence the symptom is present; however, some continuous outputs that might appear as confidence scores are often not well calibrated as actual probabilities [43].

In machine learning, the “validity” of the output is mainly evaluated by comparing it to some ground truth or criterion –often a human judgement, and increasingly LLM evaluations [44]. If the output is categorical (binary or multinomial), the metrics used are classification metrics such as sensitivity, specificity, precision, accuracy, F1 score, ROC AUC, and inter-rater reliability (i.e., between human and model scores). If the output is ordinal or continuous, then common validity metrics are mean absolute error, mean squared error, *R*^2^, and inter-rater reliability. These are performance metrics evaluating agreement with a ground truth label, which can be seen as a partial assessment, but is itself an imperfect proxy for the underlying construct (i.e., the estimand); psychometrically valid psychological assessments reflect a broader claim than performance against any single ground truth (see the section “Desirable psychometric properties of psychological assessments and how to evaluate these properties in text”).

A challenge when assessing mental health from existing retrospective text data is a person may have said “I am sad” and it is not clear how sad they are (i.e., the cardinality), since this could be a valid expression of mild and severe sadness. This ambiguity is a limitation of some static retrospective text, not of language’s expressiveness per se—in real-world dialogue, a clinician, for instance, can follow up with specific questions to resolve severity or frequency, potentially achieving even more granularity than standard rating scales. Therefore, one approach is to use text classification to try to detect whether the mention of a construct is simply present or absent in a binary way (e.g., does the text mention loneliness?). Multiple constructs that capture different severity levels in a binary way can be assessed (e.g., worry and panic).

We now describe multiple NLP methods and depict them in Fig 4, which we illustrate using classification examples. Assessing cardinality (e.g., severity) is an alternative, and adapting these methods to ordinal or continuous assessments is straightforward from an NLP perspective by changing certain elements of the model (e.g., the loss function) and evaluation metrics (e.g., see [44]). Overall, we recommend using LLMs when feasible for improved predictive performance (see the section “How to choose a natural language processing method” for an in-depth discussion), but we describe other commonly-used approaches as well.

**Fig 4:**
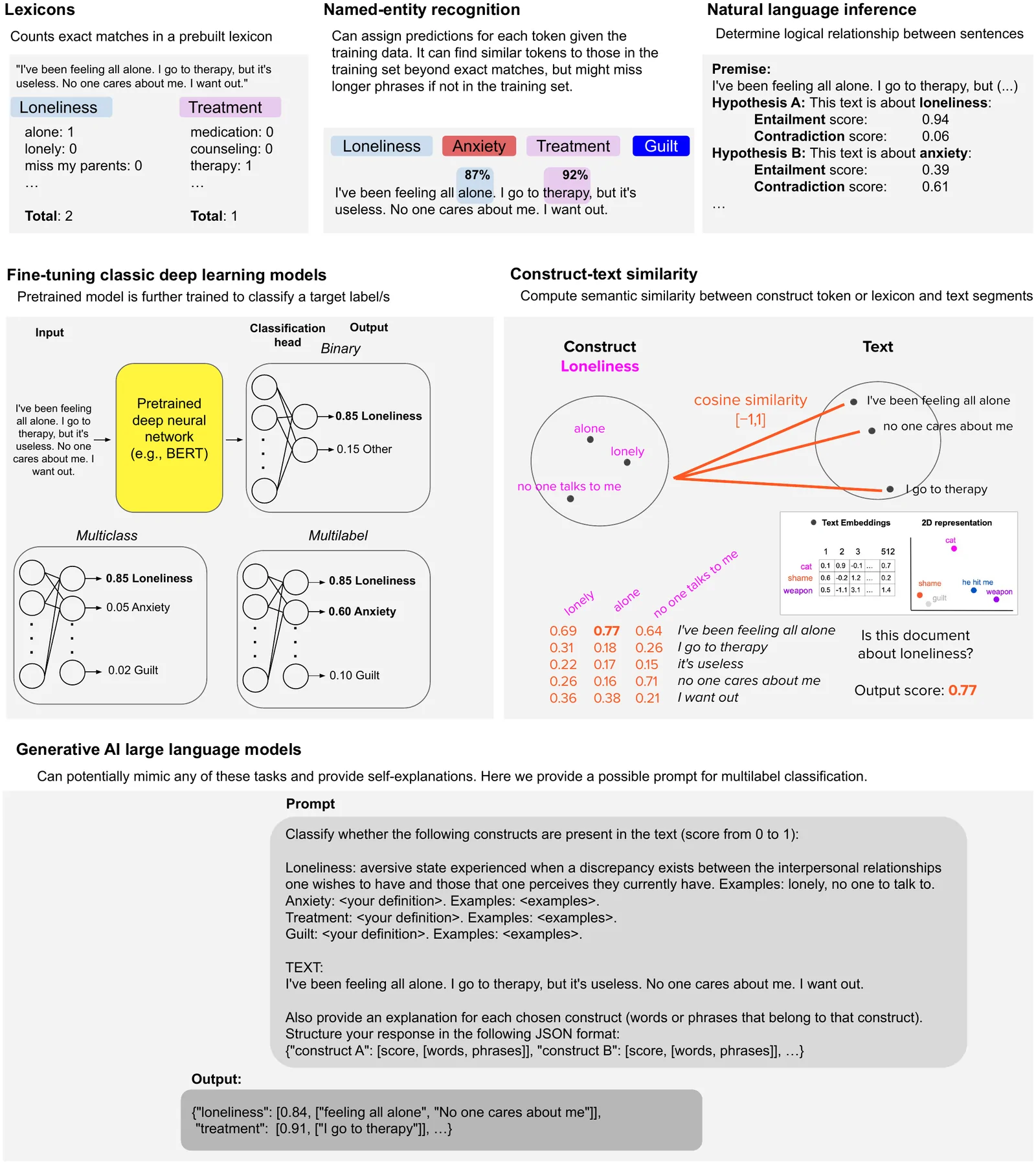
Natural language processing tasks and methods that can be used for text classification. Lexicon counts (summing all matches across many words and phrases for a given construct) can be used as is or as variables in a machine learning model to further predict a target label. The rest of the methods tend to use deep neural networks. Finetuning a model on a specific dataset (e.g., psychotherapy transcripts) that has previously been trained for the type of classification on more general data (non-mental health data) can help performance. Construct-text similarity turns words and phrases into embeddings, which are vectors in a semantic space where more similar phrases in meaning should be closer together. Here we take the maximum similarity between construct tokens from a lexicon and phrases from the target document as the final zero-shot classification score. Conceptually, this is similar to asking where does the document mention something most related to loneliness? Large language models (LLMs) tend to show state-of-the-art performance but may not be preferred due to difficulty of use or privacy limitations. Submitting many texts to LLMs can be automated through the use of LLM application programming interfaces (APIs).

These methods are best understood as combinations of choices at distinct layers of a pipeline rather than as interchangeable alternatives at a single level: (1) text representation / feature extraction (e.g., lexicon counts, embeddings, entailment scores, LLM-generated scores); (2) scoring or aggregation (e.g., summed counts, maximum cosine similarity); (3) a predictive model or decision rule (e.g., logistic regression, a gradient-boosted tree, a fixed threshold, or none in the zero-shot case, i.e., where the model is applied to a task with no task-specific training examples); and (4) the validation target against which the output is evaluated. The same tool can occupy several layers—an LLM can serve as representation, scorer, and model at once— which is why we describe methods by the pipeline stages they combine. Critically, psychometric validity is a property of the measurement outcome as a whole (the end-to-end output), not of any single internal component [45]; attributing a result to a particular component is a separate, complementary exercise that can be done by ablation (see the section “Concurrent criterion validity: Predictive performance on binary classification task of 13 types of issues” and Table 2).

**Lexicons or dictionaries.** Using lexicons is a traditional way of performing text classification [33]. Lexicons contain one or many constructs, each with an associated list of tokens (words and phrases) – which can be validated by domain experts – that are counted in a given document [35]. Higher counts indicate the construct is more likely to be present. Then these counts can be used as is or as variables in a statistical or predictive model (i.e., learning to weight specific counts to optimize prediction of some dependent variable), which would be highly interpretable and would avoid missing obvious, prototypical tokens (e.g., “anorexia” for eating concerns) that might be missed by a high-performing deep learning model [35]. Our *construct-tracker* package allows for using generative AI to automatically create the initial lexicon, which can then be validated by human experts [35]. This is useful when the goal is to detect a construct with a simple, interpretable baseline model and/or guarantee not missing certain phrases (e.g., flagging suicidal language) or as an initial screening of potentially relevant documents (For a tutorial on building or using existing lexicons, see: https://github.com/danielmlow/construct-tracker)

**Rule-based linguistic variables.** Many variables can be extracted that capture semantic, morphology, syntactic and discursive aspects of language based on rules or dictionaries (e.g., list of prepositions) and therefore do not require complex models to derive (for reviews, see [46,47]). Once extracted into variables, then a model can learn which ones characterize the presence of a given construct in text. These variables include part-of-speech tags—including the frequency of nouns, verbs, adjectives, and pronouns— and the ratio between them. A high prevalence of first-person singular pronouns (e.g., I, mine, my) can characterize speakers’ focus on others versus themselves, and a lower prevalence of nouns-verb ratio might indicate word-finding difficulties [46]. Measures of lexical richness, such as Honore’s statistic, quantify vocabulary diversity and repetition patterns. Syntactic complexity measures are derived from the length of clauses, and usage of dependent clauses, tree depth. Finally, discourse markers reflect the degree of cohesion and coherence in a speaker’s narrative; for instance, semantic coherence in discourse captures the semantic similarity between successive phrases to capture staying or going off-topic [48]. Together, these tend to capture cognitive processes (e.g., tangential speech in psychosis). They tend to not necessarily capture the mentions of psychological states such as loneliness directly but linguistic behavior that correlate with the presence of these types of constructs.

**Term Frequency–Inverse Document Frequency (TF-IDF)** is a common technique for identifying words and phrases in documents that characterize a dependent variable based on how frequently they appear in a given text relative to a larger corpus [49]. These scores can be used as variables in predictive models (e.g., see [5] for words that characterize different mental health support groups on Reddit). Unlike lexicons, TF-IDF requires no expert input, making it data-driven, scalable, and domain-agnostic. However, as with lexicons, TF-IDF treats words independently and lacks semantic understanding. Furthermore, this data-driven approach that finds words that characterize a variable is likely to be dependent on the specific dataset used and might not generalize to other datasets. Other methods presented here (including lexicons and LLMs) have the advantage of detecting a broad coverage of words that are prototypical of a construct –if designed to do so– and not just associated in some way in a specific dataset [35].

**Unsupervised topic modeling** (e.g., Latent Dirichlet Allocation or LDA, BERTopic) do not start from a deductive approach where we have certain constructs of interest like text classification does, but rather discover topics that are present in the text inductively. They group frequently co-occurring words into latent topics, which can subsequently be mapped to psychological constructs by subjectively interpreting the cluster of words or using automated methods [50]. This is data-drive and requires little preprocessing. However, one key limitation is that these approaches frequently yield low-coherence topic clusters marked by intrusions—words that are semantically unrelated to the rest of the cluster (e.g., “date” appearing among “kill,” “intrusive,” “thought”) [28,51]. Furthermore, the cluster can be interpreted as representing many different constructs. We suspect that many social scientists would prefer to begin by measuring very specifically-delineated constructs already known to be relevant to their domain (e.g., the extent to which participants discuss rumination or bullying), reserving data-driven, exploratory methods as exploratory approach aimed at surfacing potentially novel constructs. More recent embedding-based topic modeling approaches, such as BERTopic [52], attempt to improve on this by combining document embeddings (dense numerical vectors that capture meaning rather than just word co-occurrence) with clustering algorithms to group semantically similar documents before extracting representative terms for each cluster, resulting in more semantically coherent topics than LDA. However, they remain unsupervised in the sense that the resulting topics are not guaranteed to correspond to constructs of a priori theoretical interest and may be too heterogeneous.

**Supervised topic modeling** (e.g., supervised LDA, supervised BERTopic) jointly infer latent thematic structure from word co-occurrence and fit a predictive model (e.g., regression) of that structure onto an observed outcome, so the topics are shaped to be predictive of the label as well as descriptive of the text. Supervised BERTopic clusters over sentence embeddings where label information is used to warp the embedding space (via supervised dimensionality reduction) before clustering, so documents sharing a label end up closer together and more separable into distinct topic clusters. Supervision optimizes topics to predict your outcome, not to be more internally coherent, and can even reduce standard coherence scores as a tradeoff [53].

The remaining methods are generally carried out using deep learning methods. Rarely is training done from scratch on the target task (e.g., loneliness detection), given they generally require large datasets labelled for the target task; rather, pretrained models like BERT [54] are trained on a very large corpus (the entire English Wikipedia and other sources) in a self-supervised manner (e.g., predicting masked words within the input) to generate rich, internal representations that quantify aspects of meaning of words and phrases. Then this model can be fine-tuned (i.e., adapted) for a specific task (e.g., detecting loneliness in text) on a smaller, annotated dataset (e.g., documents coded for loneliness or other constructs) by attaching a feedforward neural network [55]. Pretraining can also be done in a supervised manner by training to predict labels in large, publicly-available datasets (e.g., for named-entity recognition). Then these pretrained models can be fine-tuned for the target task, which should improve performance in comparison to using the target dataset to train the model from scratch.

**Named-entity recognition** classifies each token as a possible entity belonging to a category or ontology [56]. The presence or sum of entity types in a document would return similar results as text classification. The general approach is to train a model to output a probability for each entity type.

**Natural language inference and textual entailment.** This method can be used for text classification as well [57–59]. Entailment is inferring whether two sentences are a contradiction or an entailment (e.g., “Met my first girlfriend that way.” is a contradiction of “I didn’t meet my first girlfriend until later.”; “At 8:34, the Boston Center controller received a third transmission from American 11” is an entailment of “The Boston Center controller got a third transmission from American 11.”; [60]). In a classification setup, the document to be classified can be treated as the premise for which one can build a positive hypothesis using the template “this text is about <construct > ” as well as a negative hypothesis using other labels; [57]. Gretz et al (2023) [59] fine-tune natural language inference models which output entailment and contradiction scores that serve for text classification.

**Embeddings** turn each text into a fixed-length numeric vector that captures aspects of its meaning. In supervised prediction, those vectors become the independent variables or features: texts already labeled for some construct (e.g., journal entries coded for depression severity), embed or vectorize each document with pre-trained embeddings, and train a model (e.g., logistic regression, a gradient-boosted tree) to map the embedding to the label. The model learns which directions and dimensions in embedding space are associated with the outcome (each one being relatively uninterpretable), and can then predict labels for new, unlabeled texts. Because embeddings encode semantic similarity, this can generalize better than exact-word methods like lexicons or TF-IDF (e.g., “worthless” and “unworthy” land near each other), at the cost of interpretability.

**Fine-tuning deep learning models**. Whereas traditional methods involve some preprocessing (e.g., lemmatization, data cleaning, and feature extraction), pretrained deep learning models like BERT allows for end-to-end prediction, taking raw data, skipping most preprocessing, and outputting a prediction [54]. However, this generally requires a substantial amount of labeled training data, at least in the thousands of samples (although new advances such as LoRA are improving this [61]).

**Construct-text similarity**. Since lexicons can miss important tokens that they do not have (e.g., a lexicon may have “suicide attempt” but miss “attempted suicide” or “tries to take ones life” if not in the lexicon), there is a method to find semantically similar tokens to the ones in lexicon using deep learning using relatively interpretable method called dataless classification [62], similarity-based zero-shot text classification [58] or sentecon [63], among others. We introduce a more generalized version that has more parameters to choose from for each step, which we call construct-text similarity (which we expect will be a more transparent term for psychologists), as shown in Fig A in S1 Appendix. This method works as follows: it first computes the similarity between a target construct representation (e.g., text embedding for the phrase “eating disorders” or for many tokens related to eating disorders or the average of all of these) and each token in a given document (e.g., text embeddings for phrases). It then takes a summary statistic as the final output (e.g., maximum between all construct and document embeddings). This approach produces a zero-shot output, meaning the method requires no training data. The construct can be the average of the text embeddings for a lexicon’s category, which means that it can capture much of what the lexicon captures and also similar words while a lexicon can only capture exact matches. The process of being able to capture similar words has been demonstrated to outperform lexicons [63]. It is a middle ground between interpretable lexicons which will miss similar words if not in the lexicon and standard supervised deep learning which is a black box and hard to explain. It can also combine counts (0 or N with semantic similarity if there is not an exact match). However, it does not learn the contextual cues or reasoning abilities found in the previously described deep learning models. The resulting score can be used as the output for zero-shot text classification. It was found to outperform the textual entailment approach in a zero-shot setting [58], but not when textual entailment models were fine-tuned [59]. It is also more lightweight than deep learning methods because it can run more easily on standard CPUs (central processing units, the general-purpose processors in any computer), unlike deep learning, which often requires specialized GPUs (graphics processing units, hardware built to run the many parallel computations deep learning needs). This is especially beneficial for psychology researchers who may not have the expertise to work with GPUs. (For a tutorial on construct-text similarity, see https://github.com/danielmlow/construct-tracker/blob/main/tutorials/suicide_risk_lexicon_construct_text_similarity.ipynb.)

**Large language models** that generate text can be used for text classification (i.e., generating a score) by designing prompts to score whether certain constructs are present in a given document [59,64] –which can be considered LLM-assisted qualitative coding [65,66]. (For a tutorial on using LLMs to detect constructs in text and compare to human coders, see: https://github.com/childmindresearch/llm_tracker.) However, the best-performing models (e.g., the latest GPT, Gemini, Claude) tend to be proprietary [67] and therefore require users to submit the prompt to a company’s server through a browser or API [64], which may be infeasible if the data is highly sensitive. Another reason for submitting data to APIs are because generally for a fee, users can run inference or training quickly on cloud-based GPUs (e.g., OpenRouterAI, Replicate, Huggingface API, Microsoft Azure, Amazon Bedrock). Alternatively, users can download open-source LLMs (e.g., Gemma, Llama, Qwen, Mistral series). These tend to be smaller (e.g., 2B-70B parameters) and underperform their larger proprietary counterparts (with the exception of DeepSeek R1 which has 671B parameters [68] and Llama-4 Maverick with 400B parameters [69]) and require considerable computational resources such as GPUs. Models can be quantized by reducing the precision of the weights for faster inference and fine-tuning while achieving similar performance [70] which can lead lighter models to run on a personal computer’s GPU or CPU (e.g., using packages such as transformers, Ollama, GPT4All, llama cpp), which is likely to become very prevalent as models become smaller and more efficient and hardware capabilities improve. Leaderboards include Huggingface Open LLM leaderboard (https://huggingface.co/spaces/open-llm-leaderboard/open_llm_leaderboard#/) and the Chatbot Arena (https://lmarena.ai/) [67].

### Desirable natural language processing model characteristics that can address key constraints in psychological research

Now that we have introduced several NLP methods, we highlight desirable NLP model characteristics as shown in Table 1. These tackle pragmatic constraints that psychology researchers often face. One is the limited availability of big training datasets, especially in clinical settings. This poses a challenge for fine-tuning standard supervised learning models such as BERT that require large datasets. A second challenge is the need for interpretable models to understand how a model is making predictions to increase trust that it is not biased [75]. Explainability methods also allow researchers to rank which variables are most predictive of a target variable, which can be used to advance scientific understanding of the variables or discover novel risk factors [76]. A third challenge is the need to keep sensitive data private, which currently limits the use of submitting data to cloud services to use existing proprietary generative AI models, although cloud-based data security may improve in the near future.

**Table 1:** NLP- and AI-specific model characteristics and constraints to consider when selecting a text model—specific to NLP and AI models rather than to traditional rating scales. Some are broadly desirable capabilities (e.g., zero-shot use, explainability, semantically similar matching, context-sensitivity, robustness to dataset shift), whereas others are considerations that depend on the use case rather than universally desirable features (e.g., data privacy depends on the deployment setting; reasonable errors overlaps with validity, since an error analysis bears on whether the model captures the construct, and is retained here as a practical, model-facing diagnostic).

Desirable NLP model characteristic	Description
**Zero-shot**	Traditional supervised learning requires hundreds-to-thousands of examples of text-label pairs to train (e.g., documents coded for loneliness). Self-supervised learning enables training on even larger amounts of data because it does not need to be coded and often leads to general domain models (e.g., GPT-4 onwards) that can solve different types of downstream tasks (e.g., detect loneliness in text) zero-shot, that is without any further training or fine-tuning on the target dataset. This is extremely useful to avoid the resources involved in training especially when little annotated training data is available. This is expected in the social sciences: since there are thousands of constructs that could be measured, having annotated datasets for each construct is infeasible. **NLP example:** accurately detect loneliness in text without further training (i.e., updating the weights of the model). NLP has distinguished this type of text classification problem where categories are not pre-defined as “topical text classification” versus the more standard pre-defined categories such as sentiment, genre, or spam, for which it is easier to find labeled datasets for fine-tuning; zero-shot methods are particularly useful to classify new categories [59,71].
**Explainable**	One useful definition of explainability refers to the ease with which humans can simulate how a trained model takes an input and produces an output (i.e., simulatibility [72]). This is straightforward in small decision trees (tree-structured sequence of decision rules) and linear models and less feasible in tree ensembles (models that combine many decision trees, such as random forests or gradient-boosted trees) and neural networks. Explainability should be assessed along with predictive ability: understanding what a model used for prediction is not useful if the model does not predict well. **NLP example:** Counts from lexicons tokens trained on a linear model are interpretable. For instance, why did the model output 2 for loneliness? Because it found two phrases from the loneliness lexicon. Asking the latest LLM for an explanation of its predictions can seem plausible (LLM: “I think loneliness is mentioned in this text because they say ‘no one cares about me’”), but might not be truthful with regard to the underlying prediction mechanism (e.g., some statistical and mathematical operation), which remains a black-box [73].
**Reasonable errors**	An error analysis can be performed by annotating a random subset of a model’s false positives and false negatives (or of the largest errors in a regression task) to determine whether mistakes are reasonable or not. **NLP example:** A model detecting social media users who are actively suicidal (i.e., where the user posts about a desire and/or intent to die by suicide) may incorrectly classify text about someone else’s suicide or prior suicidal ideation as a high-risk case, a more reasonable error than detecting non-mental health language as suicidal [4].
**Semantically similar matching**	Traditional keyword search methods using lexicons depend on an exact match and can miss related tokens. **NLP example:** A lexicon would not match the word “unworthy” even if it contains the word “worthless.” Deep learning methods and Construct-Text Similarity are more likely to associate them.
**Sensitive to context**	It is important that even though a model detects a certain token (e.g., “depressed”) as related to the dependent variable (depression), it should modify its prediction based on the token’s context which could include negation, hedges, degree modifiers, modality markers (e.g., “not,” “might be,” “a bit,” “very,” “I must be.”). **NLP example:** Rule-based methods such as counting tokens from a lexicon generally do not capture context (e.g., “depressed” vs. “maybe I’m depressed”) as is better achieved with deep learning.
**Ease of use & low computational resources**	Deep learning models generally require using GPUs for efficient training and for inference (when they are large) and knowledge of running models, which is infeasible for most psychology researchers. However, the use of LLMs via graphical user interfaces (e.g., ChatGPT) and cloud-based APIs (e.g., OpenRouterAI) can reduce these barriers. **NLP example:** Using lexicons in statistical models can be run on a single CPU and have an easy-to-use app, while fine-tuning or running inference for encoder BERT-style models and decoder generative AI models requires substantial technical and GPU capabilities (although light-weight models are becoming increasingly available; [70]).
**Data privacy**	LLMs can be used through cloud APIs when they cannot be downloaded because they are either proprietary or when a user prefers to avoid setting up their own GPUs and pipelines. Sending sensitive data to APIs may not be secure. **NLP example:** Sharing names, professions, addresses, numbers, birthdates, medical history, and payment information. Open-source models can be downloaded to use in a local, secure way (demonstrated in the current study).
**Robust to dataset shift**	The ability of a model trained on a given dataset to generalize to data that differs from the original dataset in time, demographic distributions, language, feature extraction methods, etc. [74] **NLP example:** Train model on data from 2021 and prove it performs well on data from 2024.

Given these constraints and NLP-model characteristics, researchers can choose different types of approaches to perform text classification, which we guide how to do in the section “How to choose a natural language processing method”. Once an NLP model is trained using machine learning, different psychometric properties can be evaluated. There is not a one-size-fits-all method, and choosing a method will depend on these constraints and on evaluating multiple psychometric properties.

## Desirable psychometric properties of psychological assessments and how to evaluate these properties in text

There are many desirable psychometric properties that we may want a psychological assessment tool to fulfill. Here we provide a concise outline of these properties with examples of how to evaluate them with text-based models. This list of properties is helpful for several reasons: first, to adapt the definitions of classical and modern psychometric concepts to fit the needs of current NLP and AI goals. And second, to inspire new types of analyses that might be less frequent among NLP researchers so they can validate their models more broadly and help link their intuitive analyses to concepts that already have a vast literature in psychometrics.

**Causal validity.** Ideally, a measurement is demonstrated to be valid when a measurement outcome (e.g., change in thermometer) is caused by an intervention on the target attribute (e.g., temperature) [45]. This causal effect can be estimated through randomized controlled trials or causal inference methods for observational data [77]. This causal perspective on validity might discard many types of so-called validity approaches listed here as necessary to prove validity; in particular, construct validity [78]. *NLP example:* evaluating the validity of an NLP model that measures state anxiety from text data: participants could be randomly assigned to a relaxation- or anxiety-inducing task and asked immediately after to write about their affect –a valid score from this NLP model would be lower after the relaxation task than the anxiety task.

**Criterion validity**. An instrument score is valid if it is associated with a criterion assumed or demonstrated to be valid [7]. The criterion is often a human or expert judgment but can also be a certain behavior or biological variable. Psychometrics has generally focused on within-sample associations, while NLP and machine learning focus on out-of-sample prediction. There are three types of criterion validity, depending on *when* the criterion and the instrument were assessed:

(a) **Concurrent validity**: The instrument’s score is associated with a more standard or previously validated questionnaire total score or questionnaire item that at face value is valid, assessed at the same time point, either through correlations or out-of-sample prediction. *NLP example:* Correctly classifying crisis counseling conversations as being about physical abuse given the counselor’s annotation (i.e., the criterion; demonstrated in the section “Study 1: assessing multiple types of mental health issues to evaluate concurrent criterion validity and content validity”).
(b) **Prospective validity**: Rating scales should be associated with future scores or future behaviors within the same person, which may require repeated measures. This will also depend on whether the construct is an episodically-changing trait or a persistent trait. Higher scores on a suicide risk assessment questionnaire should have a positive correlation with future suicide attempts. This is related to the study of dataset shift in machine learning (see Table 1). This has been traditionally referred to as “predictive validity” [7]; however, given correlation is not prediction (see Prediction entry below), we use “prospective validity” instead. *NLP examples:* (1) Demonstrate baseline language patterns are associated with future PTSD symptoms [79]. (2) Predict whether a conversation will be annotated as an imminent risk by the counselor (better assessed towards the end of the session) from the initial 25% of the conversation (demonstrated in the section “Study 2: Predicting whether to deploy an emergency service intervention for imminent suicide risk to evaluate prospective criterion validity under class imbalance”).
(c) **Retrospective validity**: Whether an instrument reflects past conditions or behaviors. For example, the score of a suicide risk assessment should correlate with lifetime suicidal thoughts and behaviors. *NLP example:* Train a model to detect language about depression and correctly identify which individuals had been diagnosed with a depressive disorder in the past, independent of current diagnosis.

**Ecological validity**. The assessment resembles real-world conditions vs. tightly controlled laboratory conditions. *NLP example:* Have participants journal by randomly prompting them during the day (i.e., ecological momentary assessment); social media data [5].

**Face validity**. Whether a test appears to be valid for members of the target population based on a subjective judgment. For instance, individuals may not support the claim that the way they draw a person –a common projective assessment– clearly reflects their personality or mental health. If a math exam is clearly too hard for test-takers, then it will have low face validity. A scale assessing honesty may purposely try to have low face validity to avoid a social desirability bias. *NLP example:* The individuals being assessed could compare the construct being assessed (e.g., anxiety) with the question they were asked (e.g., “Describe what has been worrying you the most today,” which would have high face validity versus “Describe this picture of a forest” which may have low face validity). If the model is explainable (see Table 1), they could assess the appropriateness of the explanation. For instance, if a model uses words from text like “bulimia” and “eating” to indicate eating and body image concerns but ignores “anorexia,” it may indicate low face validity [35]).

**Construct validity**. Some constructs such as pain, mood, attitudes, disability, and quality of life are not directly measurable and therefore do not have a clear gold-standard method of measurement [80]. Construct validation is thought to provide support that the measurement is measuring a given construct. A measurement score for a construct should not be highly associated with scores for unrelated constructs (discriminant validity) and associated scores for similar constructs (convergent validity). It can be demonstrated though a pairwise correlation matrix of related and unrelated scores or through prediction. Discriminant validity has a variety of methods [81]. If one of the two variables being associated is considered true (e.g., a gold standard), then the association would be a demonstration of criterion validity; when neither is, it is a demonstration of construct validity. A test can be similar to another, but they can both be invalid; therefore construct validity can be seen as an inferior form of validity than criterion validity with a gold standard, and there is good reason to suggest it does not assess validity properly [78]. *NLP example:* Among individuals at clinical high-risk for psychosis, the NLP metric of semantic coherence was correlated with positive thought disorder severity (e.g., tangentiality and derailment) and the NLP metrics of complexity (sentence length and complementizer use) were correlated with negative thought disorder severity (poverty of speech and content) [82]. A type of construct validity is **known-groups validity**: When a test score discriminates between two groups known to differ [80]. *NLP example:* A valid measure of quality of life from natural language should likely differ between groups with and without chronic pain.

**Content validity**. The systematic examination of the test content by an expert to determine whether it covers a representative sample of the behavior to be measured [83]. A scale assessing depression should cover most common symptoms; however, depression scales perhaps should be expected to have low content validity given the dozens of diverse symptoms that are considered part of depression (i.e., heterogeneity [84],). *NLP example:* Even though a classifier has relatively high performance on a test set, this does not guarantee it covers a representative sample of the behaviors of the construct being measured. A classifier can achieve higher performance detecting eating or body image concerns while missing “anorexia” if this token is relatively infrequent in the test set [35]. LIWC has low content validity for most mental health symptoms such as hopelessness or suicidal ideation. Content validity test sets compute the sensitivity of a given trained model on its ability to predict as positive a list of tokens that cover most types of instances of the construct (proposed in the current study).

**Epistemic validity and clinical utility**. Validity is often seen as a demonstration that a test measures what it intends to measure [45,85], independent of how it is being used [86]. However, a test may be used to inform a diagnosis or treatment plan or to determine the efficacy of a therapeutic or educational intervention. Aspects of how a score is interpreted and used in practice ethically or unethically are related to its epistemic validity [87] (see also [88],). Relatedly, determining whether a certain sensitivity and precision of a test is appropriate depends not on only their values but on how decisions will be made as a consequence of their values, their clinical utility. This can be quantified through a net benefit analysis which weighs potential benefits versus harms of a test, considering its true positive and false positive rates [89–91]. *NLP example:* Consider an NLP model that detects suicide ideation in the last clinical visit to determine whether their should be a follow-up; if the model has low precision, it might still lead to improved outcomes compared to not deploying it, a net benefit.

**Cultural validity** refers to the extent to which a construct, its operationalization, and its interpretation are appropriate and meaningful across different cultural contexts, reflecting both universal (etic) and culture-specific (emic) understandings of behavior and experience [92,93]. Without cultural validity, assessments risk incorporating unexamined cultural assumptions, leading to systematic mismeasurement, biased inferences, and reduced generalizability across populations. *NLP examples:* A model assessing depression from text may perform well on Western social media but misclassify culturally normative expressions of distress (e.g., somatic complaints or indirect emotional language) in non-Western or minoritized groups, indicating low cultural validity. Text-based variables highlighted as indicators of a target variable “flourishing” (e.g., autonomy- or achievement-focused language) may align with individualistic cultural values while ignoring relational or communal expressions of well-being, revealing culturally specific assumptions embedded in the model [94]. cover how text markers of depression change with race.

**Fairness**. Related to the concept of equivalence in psychometrics, a test should not unintentionally function differently for different groups (e.g., age, disability, gender, race, ethnicity, education level, sexual orientation) [7], particularly if it is unknown to the test recipient. More broadly, fairness relates to evaluating whether discoveries (e.g., five dimensions of personality) generalize as universals beyond the socioculturally-narrow populations where they were tested [95]. *NLP example:* A model underpredicts the need for an intervention for a protected group. Metrics and algorithms exist to identify and mitigate bias [96]. Discovering social bias in LLMs is another active field of research given the data used to train models, while large in scale, may also be narrow socioculturally [97].

**Measures of uncertainty**. Uncertainty can be considered as doubt about the validity of a measurement result and a dispersion of the values that could reasonably be attributed to the attribute being measured [98]. While standard deviations and confidence intervals can be estimated as average effects, individual predictions should also convey level of confidence as well as a lack of confidence when a model has not been trained on similar samples. Uncertainty methods for individual predictions include probabilities, prediction intervals, conformal sets, Monte Carlo dropout, ensembling, and Bayesian methods [99]. *NLP examples:* (1) If model predicts high risk with high uncertainty, do not deploy planned intervention and have expert inspect. (2) Generative AI models could provide confidence estimates and evidence that supports their opinions or inferences.

**Standardization**. Method by which a score’s test is interpreted by comparing a score to a representative sample (populational norms) on standardized scores (e.g., stanines, T scores; [7]). Lack of replication across studies can occur when different measurements of the same construct are done differently due to either different unstandardized research design or operationalization. *NLP examples:* (1) Leaderboards are ways of standardizing models’ criterion validity, public tokenizers can help standardize preprocessing. (2) Standards for feature extraction could be developed to rule out results across studies are not different due to differences in pipeline implementations or choices in parameters.

**Unidimensionality**. Whether the target construct to be measured is homogenous, that is, indicators measure the same thing (e.g., a specific type of insomnia) or whether it is heterogeneous and thus measures multiple things (e.g., depression [84],). The amount of dimensions can be assessed through exploratory factor analysis (EFA), categorical principal component analysis loading plots, and item response theory [100]. If items or more specific constructs have strong associations to certain latent variables and not to others, then each latent variable can be seen as a homogeneous construct. These could still be grouped into a multidimensional scale (e.g., personality, job performance). A network approach does not need to assume latent variables and can assess dimensionality through community detection of observed variables. *NLP example:* The dimensionality of a lexicon can be assessed by encoding the tokens with embeddings and clustering, where the amount of clusters (i.e., construct dimensions) would have the highest average silhouette score. Embeddings could also be used in factor analysis. Variables of a trained model could also be evaluated similarly.

**Reliability**. The consistency of a measure depends on the condition under which it is evaluated (For an extensive overview of reliability in NLP, see [9]):

(a) **Parallel-forms reliability and intra-method reliability**. Consistency of responses of a given individual to two versions of the same questionnaire that use different items [27]. *NLP example:* If we consider trained models as individuals, slightly changing prompts to a generative AI LLM can result in very different performance [21,27,101]. Testing sensitivity of a model to reinitializing random weights or parameters [27,102].
(b) **Test-retest (across time)**. Measuring same individuals using same test across time points. When this is done by a same rater over time, it is called intra-rater reliability. As with prospective validity, this will depend on whether construct is expected to change (i.e., trait) or remain stable over time (i.e., state). *NLP example:* An NLP model detecting paranoia from text had good test-retest reliability over the course of several days using intra-class correlation [11].
(c) **Internal consistency (across items)**. Degree to which items measure the same thing [103]. The most widely-used Cronbach’s alpha is not recommended as it tends to underestimate reliability due to many unrealistic assumptions, such as assuming each item on a scale contributes equally to the total score (tau equivalence) [104]. A related, older method is split-half reliability, which correlates two halves of the items (typically with a Spearman–Brown correction); Cronbach’s alpha can be understood as the mean of all possible such split-half correlations. A measurement may have high internal consistency but not be unidimensional (a more stringent concept) if items are associated with multiple latent variables (see unidimensionality section above); for instance, a test measuring both depression and anxiety may yield a high internal consistency (since these tend to be correlated), but is not unidimensional. *NLP example:* The Suicide Risk Lexicon [35] as a whole covers a diverse set of risk factors, but each of its 49 constructs could be evaluated for internal consistency by computing the average cosine similarity of each token’s embedding with a target label embedding representing the construct.
(d) **Inter-rater (across raters)**. Consistency across different raters’ independent judgements as measured through absolute percentage agreement, Cohen’s Kappa, intra-class correlation (ICC) and other methods [100]. *NLP examples:* (1) Annotators judging whether to include tokens in a lexicon [35]. (2) Two distinct topic models could be both correct because of the subjective nature of categorization, topic models could be considered raters and reliability could be assessed through Rand Index [102].

Which of these properties should be evaluated will depend on the goals of the study. Some of the properties we present may only demonstrate some expected property of a valid assessment, but not prove validity themselves (e.g., construct validity may not prove validity per se). (For further discussion on validity and measurement, see section B of S1 Appendix (“How to define measurement and validity?”).) Having said this, we expect researchers would want to evaluate most of these different properties depending on their application, but might only evaluate some of them (e.g., standardization) after more fundamental evaluation (criterion validity) is assessed.

### Assumptions of text data for psychometric analysis

Traditional psychometrics built on reflective latent-variable models assumes ordinal or continuous item responses, local independence, and one or more common factors. These are properties of that measurement model, not of text data per se, and they bind only when such a model is fit to text-derived variables (e.g., embeddings used in factor analysis; The section “Other psychometric methods”). Most of the framework above instead concerns supervised classification, whose relevant assumptions are those of predictive modeling: that the training sample is representative of the deployment population, that generalization is evaluated out-of-sample, and that the text–label relationship is stable across time and population (or that shifts are modeled; see dataset shift, Section 3.2). Given this, three clarifications follow. First, both structured elicitation (e.g., “Describe what has worried you most today”) and unstructured or social-media text are admissible; structured text raises the probability the construct is expressed and improves face and content validity, while unstructured text can raise ecological validity at the cost of control over whether the construct surfaces. Second, when a single text expresses multiple constructs (e.g., both anxiety and resilience), we do not partition variance across latent dimensions; constructs are scored independently through multi-label classification or separate binary models, so a document can score positive on several at once. Whether co-expressed constructs are correctly distinguished is then a discriminant-validity question that our content-validity test sets address directly, by checking that a construct’s prototypical tokens are detected by that construct’s model and not by others. Third, as noted in The section “Brief overview of NLP methods for psychological assessments”, the document is the scored unit; there is no fixed scale-item analogue, and the prototypical tokens of our content-validity test sets serve as a separate diagnostic notion of item.

The following two studies we performed demonstrate trade-offs of selecting a given NLP method as a function of the dataset, goal, and psychometric property that should be fulfilled. The studies try to assess variables related to psychological crises and mental health, tasks that highlight the need for models that are valid, explainable, and keep sensitive data private. In study 1, we compare predictive performance of a wide variety of NLP methods. Furthermore, we demonstrate why it is important to evaluate content validity, and we provide a novel way of doing so: evaluating whether a model trained to detect a construct can correctly classify highly prototypical and obvious words and phrases of said construct, as defined by experts. In study 2, we showcase a common scenario of class imbalance where the outcome of interest is rare. We predict the target variable –which is in the future– using increasingly larger segments of the crisis counseling conversation (i.e., prospective validity). In this high-stakes scenario, we use an interpretable method to avoid unknown bias and to understand which symptoms most predict future risk. In neither study, do we attempt to achieve a fully psychometrically validated model, which is beyond the scope of this study. We wish to simply showcase how to test different NLP models and evaluate a few psychometric properties of these models. Certain properties would require additional measures (e.g., convergent and discriminant validity) or repeated measures (e.g., test-retest reliability), which we do not have in these datasets.

## Study 1: Assessing multiple types of mental health issues to evaluate concurrent criterion validity and content validity

Here, we present a use case to demonstrate how to train and validate these different types of NLP methods to detect a diverse set of psychological constructs in two datasets: (a) issues discussed during de-identified Crisis Text Line conversations, as annotated by the crisis counselor immediately following the conversation; and (b) similar types of issues from Reddit (described in the section “Datasets”). The semantic variety of issues –from suicide to bullying to gender/sexual identity– should allow us to evaluate the sensitivity of the models to these different types of constructs. Demonstrating that an NLP model can detect what counselors coded as the conversation happened is a form of concurrent criterion validity (that is, if we consider their judgment ground truth). However, our main goal is to demonstrate that while being able to predict a human-annotated label is key, high-performing models may or may not be capturing a representative set of expressions of the target construct (i.e., low content validity). To assess this, we introduce a novel way of evaluating content validity (see the section “Evaluating content validity with content validity test sets”).

### Datasets

**Crisis Text Line.** Crisis Text Line is a nonprofit organization that provides free, 24/7, confidential mental health support for individuals in crisis who can begin a conversation with a trained, volunteer counselor via text messaging, WhatsApp, or webchat. Crisis Text Line users have been reported to be predominantly young (76% under the age of 25), female (79%), white (53%) and heterosexual (52%) [105]. We built several different datasets using Crisis Text Line data, containing de-identified crisis counseling conversations between individuals in crisis and counselors from the years 2017–2022. Years 2020 and 2021 were omitted to avoid symptoms and concerns that were prevalent during the Covid-19 pandemic. In Fig 5 we show the distribution of the 13 most common types of issues as labelled by the counselors. We built balanced binary datasets (training set N = 4000, test set N = 600) where only one of these issues was present vs. a random selection of the remaining types of issues from a total of 52,270 unique conversations. We balanced the test set as well so that comparisons across methods and constructs reflect model’s ability to tell the two classes apart rather than differences in base rate; we evaluate performance under a realistic, imbalanced class distribution in Study 2. Binary models were used because multi-class classification could change radically depending on what constructs are chosen and we want to reflect a psychometric test that results in a single score. The sample size was chosen to test how increases in training set affects performance and we consider 4000 samples would be among the larger samples in clinical studies.

**Fig 5:**
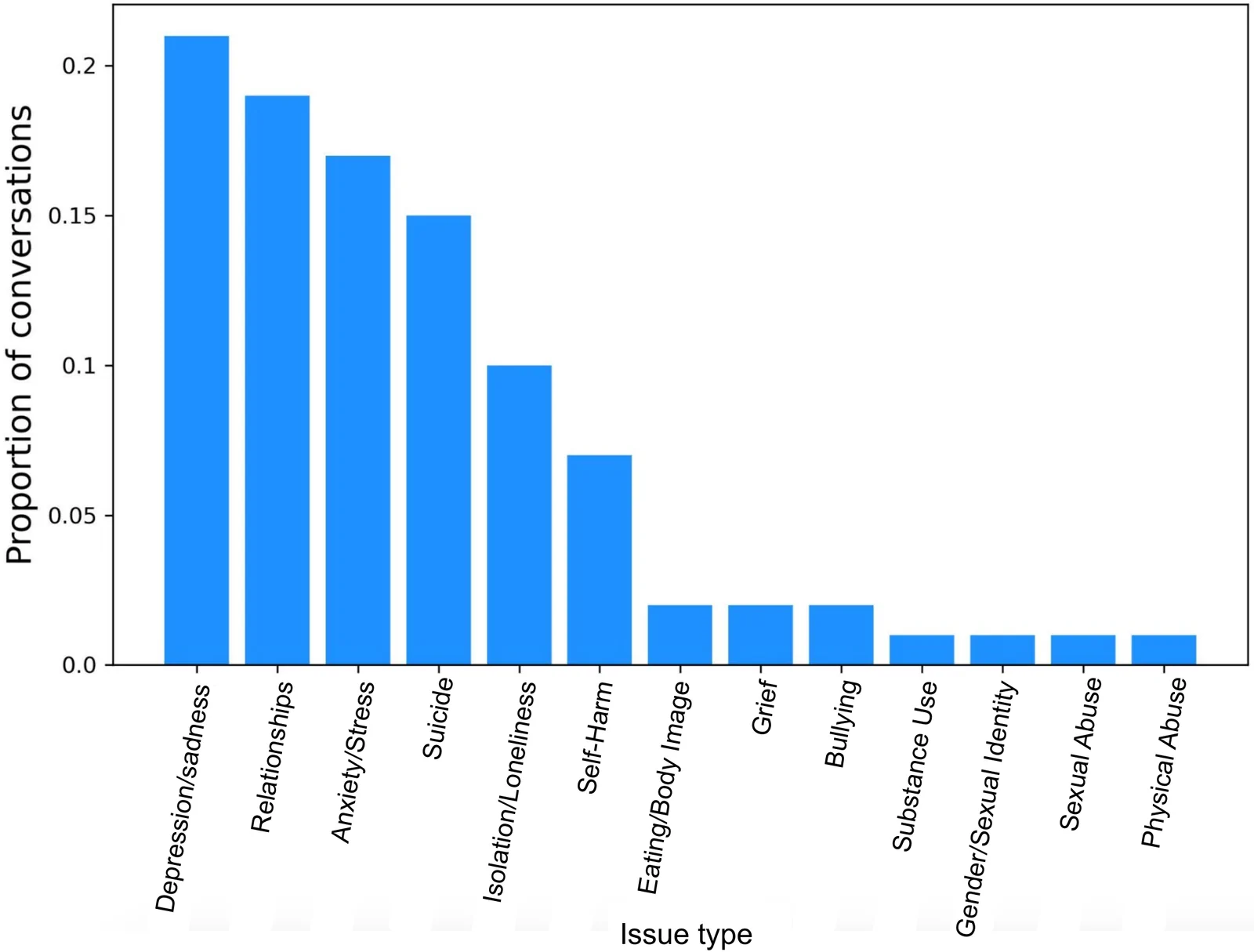
Distribution of types of issues.

**Reddit**. Reddit is a widely-used forum, used by 22%, of U.S. adults [106]. It is organized into subreddits, many of which are lightly-moderated spaces for seeking and providing mental health support. Using the Python praw package (v7.8.1), we downloaded posts from 11 subreddits (4000 for training and 600 for testing for each subreddit), reflecting most of the Crisis Text Line issues (i.e., Depression, Anxiety, SuicideWatch, Lonely, SelfHarm, EatingDisorders, GriefSupport, Bullying, Addiction, AskLGBT, SexualAssault). Since these are public data, we can evaluate the performance of proprietary state-of-the-art LLMs in their ability to identify the subreddit of each post. Posts were from 2025, after each model’s training data cutoff date, so these samples were not in the model’s training data. This also allows us to evaluate how much our results change across two datasets, keeping constructs relatively constant. This dataset differs from Crisis Text Line in being a monologue (looking at just the post content), not a dialogue, and coming from a different population: while Reddit users also tend to be young, they are characterized by being less young (44% under 29 years old), mostly male, and with more Asian and Hispanic than White users [106]. Real world use for detecting subreddit of a post include: discovering language patterns and symptoms belonging to a mental health community and assigning posts to optimal subfora (if this were required).

### Models

We include interpretable methods (lexicons, similarity-based text classification) given psychologists commonly use models for high-stakes decisions or to advance the understanding of what predicts a given construct. And we also include zero-shot methods (construct-text similarity and generative AI) given the previously discussed challenges of obtaining computational resources and the moving target of constructs that might need to be assessed.

**LIWC-22 lexicon.** LIWC is a lexicon that counts the proportion of tokens related to 117 categories including semantic constructs (family, anger) and nonsemantic linguistic variables (1st person pronouns, unique words).

**Suicide Risk Lexicon (SRL).** this lexicon contains tokens (words and phrases) associated with 49 different risk factors for suicidal thoughts and behaviors (STBs) including direct self-injury, hopelessness, and discrimination [35]. It has been shown to surpass LIWC and offers higher content validity (i.e., more related constructs) than LIWC for language around psychological issues and imminent risk. Initial tokens were built using GPT-4 turbo and later validated by clinicians. Counts are normalized by document word count. For a tutorial, see: https://github.com/danielmlow/construct-tracker/blob/main/tutorials/suicide_risk_lexicon.ipynb.

LIWC-22 and SRL were trained using logistic regression. Hyperparameter tuning was done using Bayesian optimization using the *scikit-optimize* package on a 5-fold cross-validation of the training set. Hyperparameter tuning of the Ridge alpha parameter included values [0.001, 0.01, 0.1, 1, 10, 100].

**Construct-Text similarity (CTS).** We apply this method, as described in the section “Brief overview of NLP methods for psychological assessments”, twice: once by building the construct representation using only one prototypical token and again by using all prototypical tokens from the suicide risk lexicon as construct’s tokens; prototypical tokens are those that scored 3 out of 3 by all clinicians in how much they represent a given construct (e.g., “anorexia” is very prototypical of eating disorders while “didn’t eat much” is less so). We tokenize the document into independent clauses (with subject and predicate) to capture more minimal units of meaning than sentences separated by punctuation. We compute the maximum similarity to capture the document token with the highest similarity to any prototypical lexicon token. We then min-max scale the output of the cosine similarities between 0 and 1 to match the scale of other models.

**Open-source LLM Gemma instruct models.** Gemma 7B is an open-source generative LLM that was high-performing on multiple tasks involving reasoning, question-answering, math and science, and coding at the time of analysis [107]. Gemma 2B is a smaller version that is expected to underperform but can run efficiently on a personal CPU unlike its larger counterpart. These models were downloaded onto our private and secure computer cluster. We provided the prompt displayed in Fig B in S1 Appendix. We did inference using Gemma 2B-it on a single NVIDIA GeForceGTX1080Ti GPU for 10 hours and Gemma 7B-it on a single QUADRORTX6000 for 16 hours.

**Proprietary LLM Google Gemini 2.0 flash & OpenAI GPT-4o models**. These models are among the highest performing models at the time of analysis (https://huggingface.co/spaces/lmarena-ai/chatbot-arena-leaderboard). Therefore, we used these for public Reddit data but not Crisis Text Line data. They also allow for a prompt that includes the entire codebook which reduces API requests considerably (see Fig C in S1 Appendix). We randomized the order of the constructs in the prompt for every submission to avoid an ordering bias. To encourage the model to select the most highly predicted tokens, the temperature—a parameter controlling output randomness or creativity that typically ranges between 0–1— was set to a low value of 0.1. If the output of LLMs did not contain a parseable JSON (approximately 3% of cases), random output scores (i.e., between 0 and 1) were generated.

### Concurrent criterion validity: Predictive performance on binary classification task of 13 types of issues

In Table 2 we show the average and range of the performance of the different binary text classification models across the different types of issues in two datasets, summarized using ROC AUC, the probability that a randomly chosen positive instance will be ranked higher than a randomly chosen negative instance by the classifier (0.5 = random; 1 = perfect prediction). In both datasets, the SRL outperforms LIWC, which is expected given it actually captures more relevant constructs to mental illness (i.e., as higher content validity at face value). In the Crisis Text Line dataset, the CTS model—using just a single variable in a zero-shot fashion– surpasses LIWC and the Suicide Risk lexicon trained on 300 documents and matches LIWC trained on 4000 samples. In the Reddit dataset, the CTS models performed relatively well: they only marginally underperformed the lexicon models but used only one variable and did not require any training (i.e., zero-shot). The LLMs require no additional training data from the target dataset (e.g., Crisis Text Line, Reddit) and improve their performance likely as a function of parameter size and recent innovations in training which allow for larger training datasets (while model and training specifications are proprietary, GPT4o and Gemini 2.0 flash are likely larger than gemma 2–7b models; [108]).

**Table 2:** Performance of different types of text classification methods. Number of variables are shown in parentheses. Zero-shot models require zero additional training data and are therefore displayed separately. The models that performed best on the test set (highest ROC AUC) and on the content validity test sets (highest sensitivity of prototypical tokens) are highlighted in bold. Only public Reddit data was submitted to proprietary APIs (Google, OpenAI). This design also functions as an ablation across pipeline layers: the feature set (LIWC-22 vs. SRL vs. construct-text similarity) varies orthogonally to training-set size (0/100/300/4000) and to the model, and construct-text similarity is reported with a single prototype vs. multiple prototypes—allowing readers to attribute a change in performance or content validity to the representation, the amount of training data, or the model. SRL: Suicide Risk Lexicon; CTS: Construct-Text Similarity; single: single prototypical token; multi: multiple prototypical tokens.

Models (N variables)	Train set	Crisis Text Line		Reddit	
		ROC AUC mean [min–max]	Content validity mean [min–max]	ROC AUC mean [min–max]	Content validity mean [min–max]
LIWC-22 (117)	100	0.69 [0.58-0.77]	0.80 [0.38-1.00]	0.80 [0.73-0.89]	0.86 [0.56-1.00]
LIWC-22 (117)	300	0.75 [0.66-0.84]	0.74 [0.21-1.00]	0.84 [0.78-0.90]	0.82 [0.62-1.00]
LIWC-22 (117)	4000	0.82 [0.73-0.89]	0.77 [0.40-0.96]	0.90 [0.85-0.94]	0.88 [0.70-1.00]
SRL (50)	100	0.74 [0.66-0.79]	**1.00** [0.98-1.00]	0.84 [0.73-0.95]	**0.98** [0.86-1.00]
SRL (50)	300	0.80 [0.69-0.87]	**0.97** [0.67-1.00]	0.88 [0.81-0.94]	**0.96** [0.78-1.00]
SRL (50)	4000	**0.86** [0.73-0.95]	**0.99** [0.91-1.00]	**0.93** [0.86-0.98]	**0.98** [0.78-1.00]
CTS single (1)	0	**0.82** [0.59-0.95]	0.51 [0.16-0.79]	0.79 [0.65-0.89]	0.43 [0.13-0.69]
CTS multi (1)	0	**0.82** [0.71-0.92]	**1.00** [1.00-1.00]	0.83 [0.71-0.92]	0.84 [0.53-0.97]
gemma-2b-it	0	0.61 [0.41-0.79]	0.92 [0.40-1.00]	–	–
gemma-7b-it	0	**0.82** [0.62-0.90]	0.91 [0.50-1.00]	–	–
Gemini 2.0 flash	0	–	–	**0.91** [0.83-0.95]	**0.91** [0.78-1.00]
GPT-4o	0	–	–	**0.91** [0.83-0.96]	**0.94** [0.85-1.00]

### Evaluating content validity with content validity test sets

To evaluate content validity quantitatively (albeit in a simple way), we introduce content validity test sets: lists of tokens judged by experts to be highly prototypical of the construct of interest. Prototypical tokens are those that first come to mind when thinking of a construct (“I’m a burden” or “I’m a nuisance” for burdensomeness) –given a specific definition– and have fewest variables in common with other constructs [35]. Prototypical tokens should not lead to many false positives where the person is not expressing the construct (e.g., “I’m anxious” for burdensomeness). While these tokens are likely to appear in a large enough test set, their relative frequency will be different, as depicted in Fig 6. A random test set will evaluate a model’s ability to generalize to real, unseen data; while a content validity test set will answer the question: how many of the obviously prototypical tokens can the model detect? These lists can be obtained from publicly available lists made by experts (e.g., symptoms in DSM-5) or more rigorously by creating a lexicon and validating it with clinicians, as done with the Suicide Risk Lexicon (SRL; [35]) (We specifically used SRL tokens judged 3/3 by experts (srl_prototypes_v1-0) from construct-tracker package. For a tutorial, see: https://github.com/danielmlow/construct-tracker/blob/main/tutorials/suicide_risk_lexicon.ipynb). We would expect high-performing models to capture all prototypical tokens, especially those scored 3 out of 3 in clinician evaluations of prototypicality as we test here. Therefore, the metric of choice is sensitivity: out of the prototypical tokens how many can the model correctly predict? While traditional test sets include non-obvious positive cases making a perfect prediction of 1 very difficult, we expect all models to have a perfect sensitivity close to 1 in detecting obvious tokens, especially those built using SRL (SRL and CTS). These content validity test sets provide a model-agnostic method of evaluating content validity, which can also serve as an explainability tool (i.e., what information does the model capture?) [26]. evaluated content validity of text embeddings through probing experiments: text is encoded with a given embeddings model *x* (e.g., BERT) and with an interpretable feature *f* (e.g., sentence length); the embedding is assumed to contain the interpretable information if it can predict it, a common approach in explainable machine learning. However, this does not answer whether a model captures a representative set of prototypical expressions, which may be closer to the definition of content validity, and we try to demonstrate through our complementary approach, which unlike probing is dataset-independent and works for evaluating models and not just embeddings.

**Fig 6:**
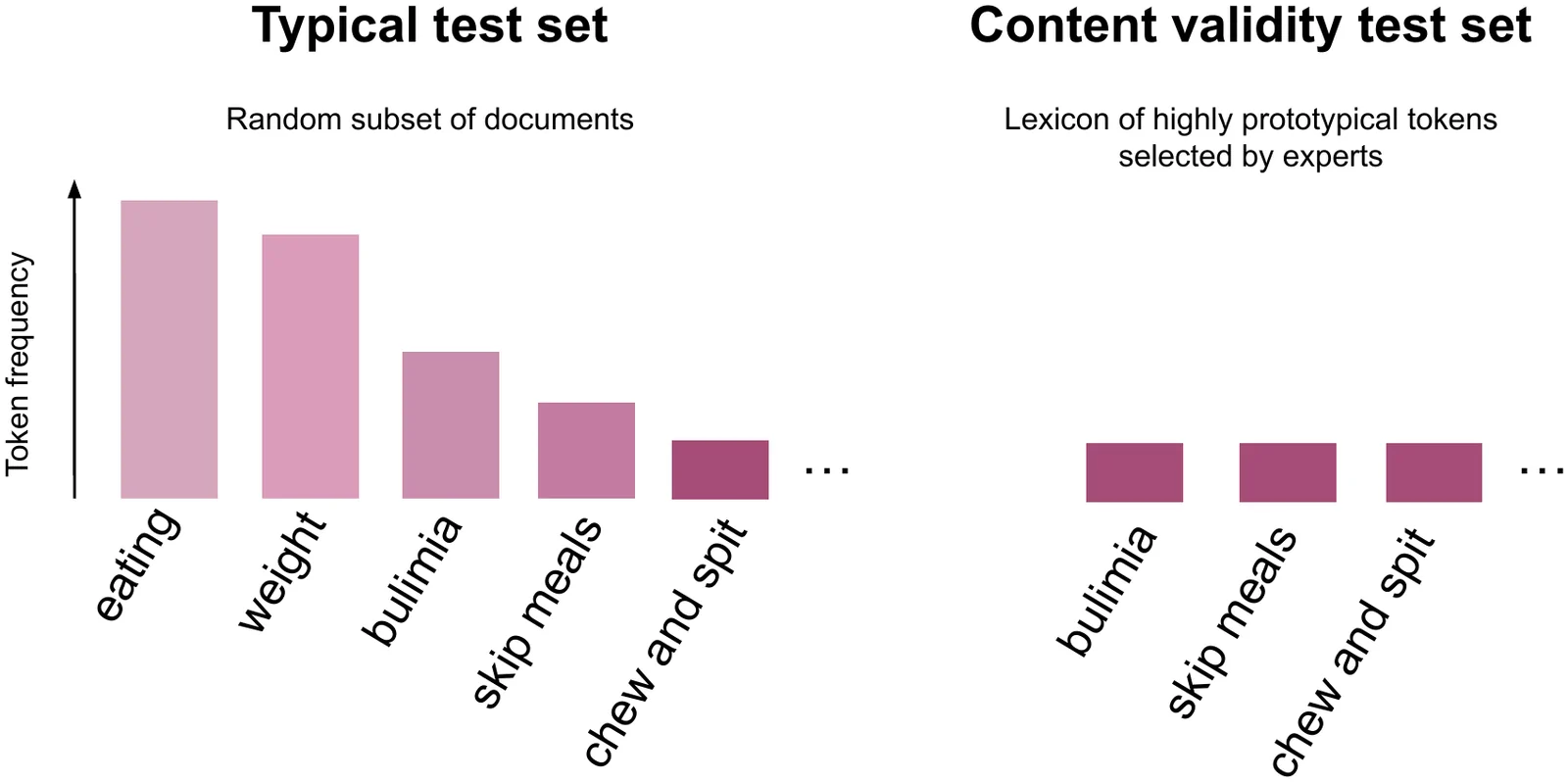
Typical test sets might devalue important but infrequent expressions of the target construct. Typical test sets influence models to use tokens to improve prediction to the extent they are associated with the target construct. They test generalizability to unseen data. Certain infrequent tokens could be ignored and still achieve high performance. On the other hand, content validity test sets are lexicons of prototypical words and phrases related to the construct as validated by experts, where all tokens have equal weight to evaluate whether a model detects a good coverage of expressions of a construct. Prototypical tokens are expected to be both sensitive and specific by experts (i.e., “eating” would create many false positives). Individual tokens should be detected as positive by a model that claims to detect the target construct (i.e., high sensitivity of these tokens). This is a proof-of-concept on how to evaluate content validity of text models. This can also be evaluated by placing tokens in multiple sentences each, generated using LLMs. We use the most prototypical tokens (3/3) from relevant constructs of the Suicide Risk Lexicon [35].

In Table 2 we show the average and range of the performance of the same binary text classification models as the section “Concurrent criterion validity: Predictive performance on binary classification task of 13 types of issues” but now tested on lists of clinically-validated tokens that are highest on prototypicality (i.e., content validity test sets), computing the sensitivity for these tokens (i.e., content validity). Any misses are considered an obvious mistake. As expected, SRL-based models (SRL and CTS using prototypical SRL tokens) are able to capture the prototypical tokens except CTS (1 prototype) which has low performance. Evaluating the model just on out-of-sample prediction using metrics such as ROC AUC or F1 score (i.e., criterion validity) miss this lack of content validity. Surprisingly, gemma models perform extremely well on average, although much worse for some constructs. Unsurprisingly, the more recent and larger Gemini 2.0 flash and GPT-4o models perform very well on average.

### Summary of Study 1

In summary, from a Text Psychometrics standpoint, Study 1 shows that criterion validity and content validity can dissociate: several methods reached good criterion validity (ROC AUC > 0.75) on the held-out task yet missed many prototypical expressions of the construct (content-validity sensitivity < 0.80), a gap that a single performance metric would hide. The lexicon built for this domain (SRL) outperformed the general-purpose lexicon (LIWC) and, together with construct-text similarity using multiple prototypes, captured nearly all prototypical tokens, whereas construct-text similarity with a single prototype had high criterion validity but low content validity. The practical lesson is that evaluating only out-of-sample prediction is insufficient; our content-validity test sets provide a model-agnostic, dataset-independent way to check whether a model covers the range of a construct’s expressions.

## Study 2: Predicting whether to deploy an emergency service intervention for imminent suicide risk to evaluate prospective criterion validity under class imbalance

The goal of this second use case is to present a more challenging NLP task: predicting a low-base rate phenomenon: whether the outcome of the conversation resulted in requesting an emergency services intervention, resulting in an imbalanced dataset. Furthermore, we will predict this outcome using earlier parts of the conversation, which is a form of prospective criterion validity. We show how different classification metrics improve as more messages are included. We finish by providing an algorithmic explanation (i.e., feature importance), that is, revealing which risk factors are most predictive of the outcome which can help inform theory.

### Dataset

During Crisis Text Line conversations analyzed, counselors assessed for suicidal thoughts, suicidal plans, access to means, and imminent 48-hour timeframe, in that order (see [35] for more details). If a texter is determined to be at a high risk by the crisis counselor, the counselor will involve a supervisor. The supervisor evaluates whether the texter is in immediate danger and unable to create a safety plan or deescalate, in which case the supervisor may contact local emergency services. To better dissociate the likely highest risk group in the dataset, we created two groups: (a) suicidal desire without plans, means, or timeframe, and (b) cases where an emergency service intervention was initiated, which has all symptoms present and cannot be fully deescalated. Such models could speed up risk assessments (e.g., alert the supervisor earlier), give a second opinion to the counselor and supervisor, or, after appropriate validation, be used in a different setting where there is no counselor (e.g., social media). We balanced the training set by downsampling suicidal desire cases to the imminent risk sample size to avoid the model over-predicting values closer to the majority value, resulting in a training set of 3500 (half in each group). The test set included a total of 2358 cases, with 2152 suicidal desire cases without plans, means, or timeframe cases (91% of test set) and 206 emergency services intervention cases (9% of test set).

### Model

For this task, we wanted an explainable model to improve trustworthiness and so we could perform feature importance analysis to understand which risk factors are associated with imminent suicide risk needing an emergency service intervention. We chose CTS with all prototypical tokens given its high performance in the prior task in capturing constructs. We used all constructs from the Suicide Risk Lexicon as independent variables and trained a LGBMClassifier model. Hyperparameters [and values] tested for the LGBMClassifier were: num_leaves [30,45,60], colsample_bytree [0.1, 0.5, 1], max_depth: [0,5,15], min_child_weight: [0.01, 0.001, 0.0001], min_child_samples: [10,20,40].

### Prospective criterion validity: Predicting whether to deploy an emergency service intervention for imminent suicide risk

In Fig 7 we report predicting the outcome of the conversation resulting in an emergency service intervention as the conversation advances (computed on the quartiles of the number of messages across all participants of the training set: 25% = 28, 50% = 43, 75% = 64, 100% varies by conversation). Similar performance to using the entire conversation can be achieved with the first 3/4 of the conversation, which could help speed up potential alert systems that could be designed in the future, if the number of messages or duration of conversation could also be reliably predicted, to help supervisors make a decision. Whether this model is clinically useful (one of the psychometric properties we described), that is, whether these classification metrics are sufficiently high for a model to be deployed –in particular, models with a low precision, which is common in low-base rate phenomena such as suicide risk assessments [90]– could be evaluated quantitatively with a net benefit analysis, which should include weighing potential benefits versus risks of harm of emergency services interventions, which may vary across populations [89–91].

**Fig 7:**
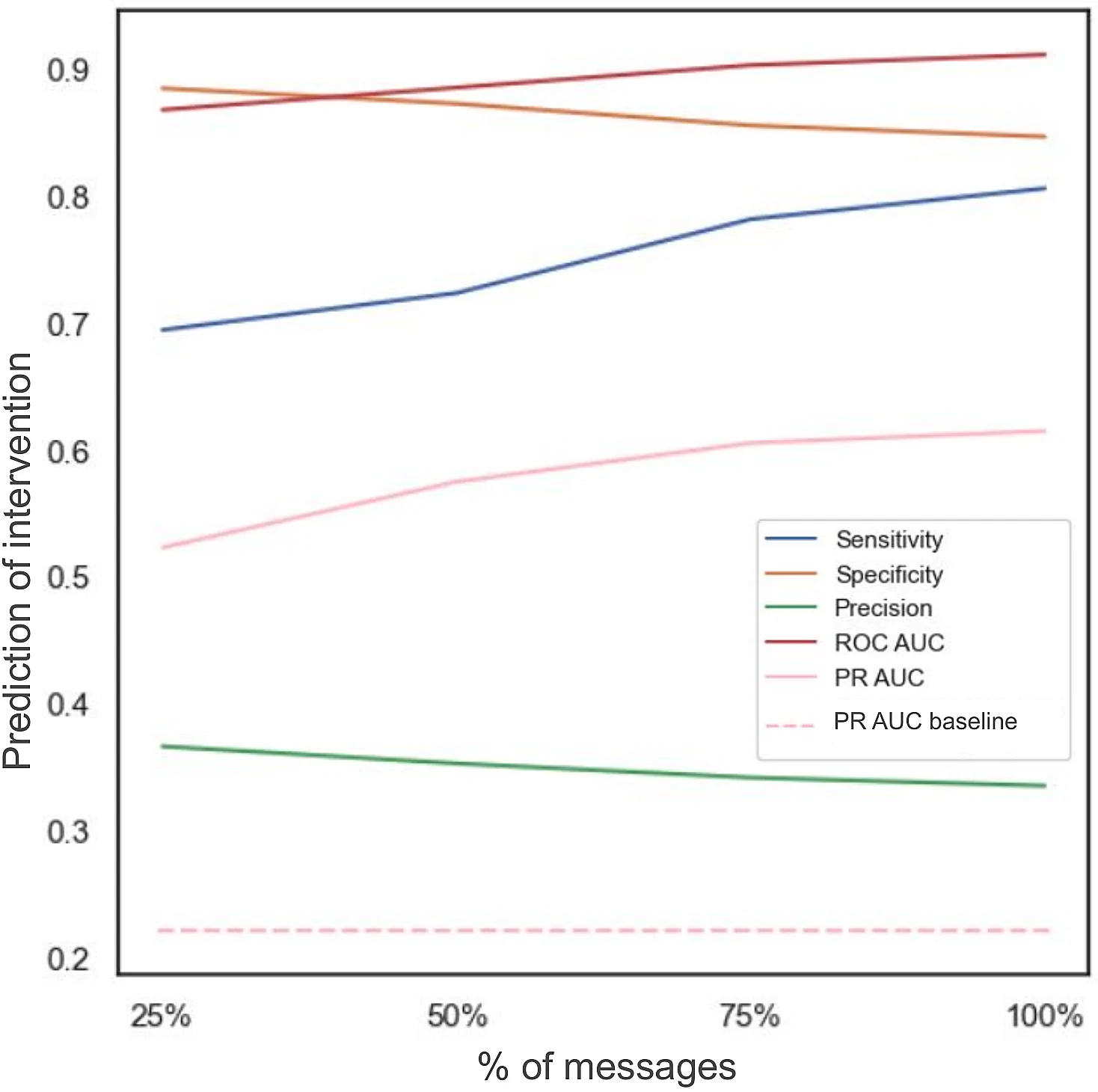
Predicting a request for an emergency service intervention (decided towards the end of the conversation) as the conversation advances. These metrics have different reference (chance) points and should not be compared by vertical position: ROC AUC is relative to 0.5, whereas PR AUC and precision are relative to the outcome base rate (dashed line, ≈0.22), and sensitivity and specificity have no fixed chance level individually. The key result is the trajectory — performance as a function of how much of the conversation is observed — rather than any single metric’s absolute height. Because both false positives (e.g., premature escalation) and false negatives (mitigated here, since the exchange with a counselor continues) carry costs, one can take a balanced view and highlight PR AUC relative to its base-rate baseline as a summary that reflects the rare positive class. Relative to the base rate of 0.22 (dashed line), PR AUC is roughly 2.4 × the baseline even at 25% of messages (≈0.52) and rises to 2.8 × the baseline by the full conversation (≈0.62).

### Feature importance

Understanding which variables are associated with suicide risk from a language perspective can help inform the theory of suicide risk: what risk factors should counselors pay particular attention to? To compute feature importance with the LGBMRegressor model, we calculated the improvement in the information gain (i.e., the splitting criterion) from using a given feature in a tree’s split (i.e., how much a given feature helps to reduce the error). Other methods include SHAP [109], leave-one-feature out (especially when there is colinearity) (https://github.com/aerdem4/lofo-importance), and permutation feature importance.

In Table 3 we show the top 20 variables for predicting imminent risk with emergency service intervention. Mentions of lethal means for suicide (e.g., cutting), Other substance use & overdose (“overdose,” “pills”), and having the texter write more than the counselor were the most predictive variables for detecting the need for an emergency service intervention.

**Table 3:** This interpretable method with high content validity allows us to report which known suicide risk factors from the literature are most predictive of an emergency service intervention scenario. Importance: feature importance, which is operationalized as the improvement in the information gain from using a given feature in a tree’s split. Values are interpretable only in relative terms: larger numbers indicate a more predictive feature, and a feature with roughly twice the value contributed roughly twice the total gain. For example, Lethal means for suicide (5106.62) contributed about 1.7 × the gain of Other substance use and overdose (2964.54) and roughly 28 × that of Hopelessness (179.73).

Feature	Importance
Lethal means for suicide	5106.62
Other substance use and overdose	2964.54
Texter-counselor word count ratio	1951.01
Police & incarceration	830.73
amount of messages counselor	688.64
Depressed mood	624.06
Anxiety	487.35
Anhedonia & uninterested	422.76
Panic	406.98
Borderline Personality Disorder	378.76
Impulsivity	341.36
amount of messages texter	341.35
Passive suicidal ideation	339.31
Rumination	296.11
Bipolar Disorder	295.19
Agitation	258.65
Entrapment & desire to escape	250.47
Active suicidal ideation & suicidal planning	235.09
Other suicidal language	190.90
Hopelessness	179.73

### Summary of study 2

From a Text Psychometrics standpoint, Study 2 demonstrates prospective criterion validity under realistic class imbalance: an interpretable pipeline (construct-text similarity features from the Suicide Risk Lexicon feeding a gradient-boosted model) predicted a future emergency-service intervention from only the first portion of a conversation, with performance improving as more of the conversation was observed and near-complete performance reached by three-quarters of the conversation. Because the outcome is rare (9% of the test set), we report precision and PR AUC alongside ROC AUC, and note that whether such a model should be deployed is a question of clinical utility (net benefit) rather than discrimination alone. The interpretability of the method also let us recover which known risk factors were most predictive—lethal means, substance use and overdose, and the texter writing more than the counselor— showing that a psychometrically-motivated, explainable model can both predict and inform theory.

## Discussion

We have introduced Text Psychometrics as an important area of research to improve the validity and reliability of NLP methods being developed for psychological assessments in text data. We reviewed how to validate models that use NLP to assess psychological constructs in text through examples and through two original studies. In particular, we have provided a novel method for evaluating content validity using lexicons and expanded a similarity-based text classification method which we call construct-text similarity which is interpretable and lightweight. Next we help guide researchers in choosing the optimal NLP method given their research and dataset constraints.

### How to choose a natural language processing method

In Fig 8 and in the following discussion we provide a practical decision guide summarizing our qualitative analysis of the trade-offs of different NLP methods, as a function of different, key desirable properties.

**Fig 8:**
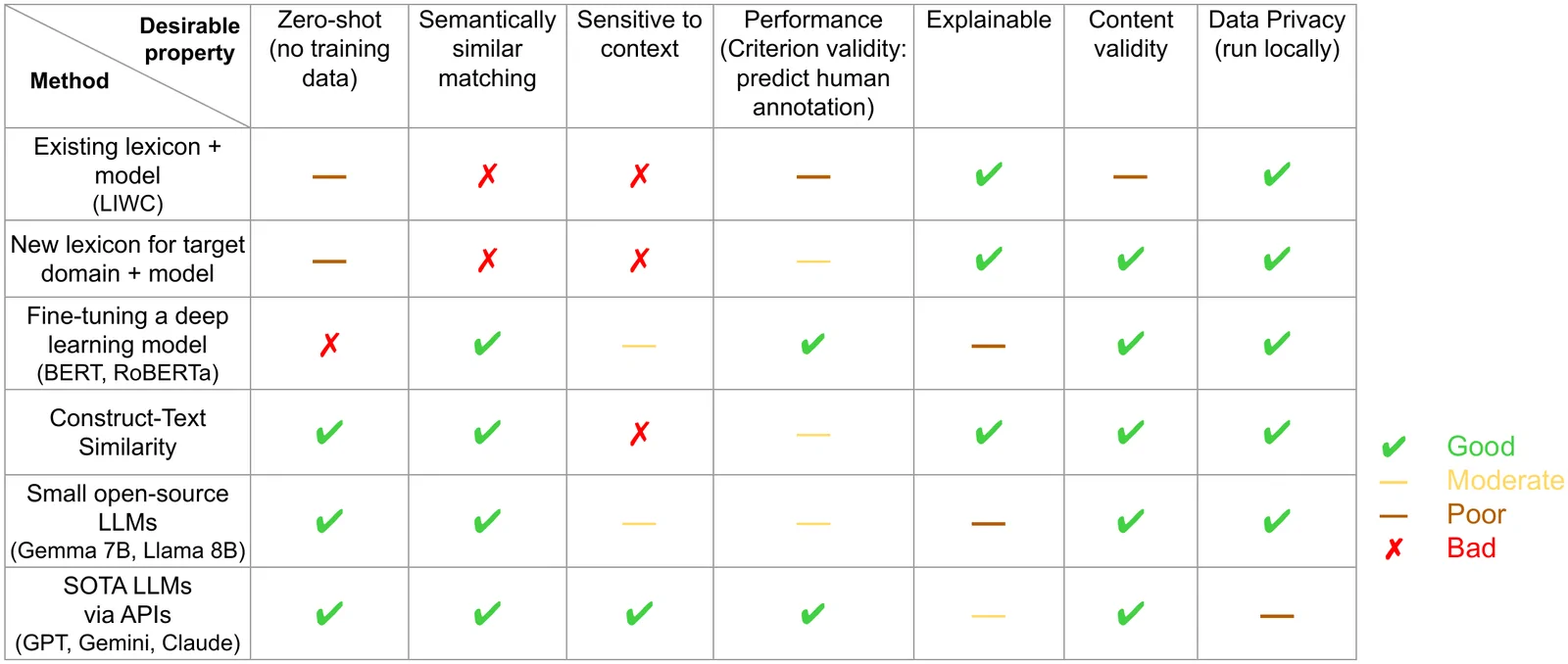
Desirable properties and how different types of NLP methods tend to compare. LLM: large language model. SOTA: state-of-the-art (outperforming other models on many NLP benchmarks). Each property bears on specific pipeline layers: criterion validity (performance) evaluates the end-to-end output; content validity primarily reflects the representation-plus-scoring layers (coverage of prototypical expressions); explainability reflects the model layer; and data privacy reflects the deployment setting rather than any single component.

The first point to make is that using an existing lexicon is rarely the optimal choice given it generally has lower predictive performance compared to other methods due to lack of incorporating contextual information and/or reasoning capabilities. We also argue it tends to lack content validity at face value since it does not generally capture the main target constructs of interest in any given study [35]; furthermore, when used to detect a given construct, the resulting model misses many prototypical tokens of the construct as shown with the content validity test sets (Table 2). Nevertheless, an advantage of using a widely-used lexicon like LIWC is to compare to prior findings so as to improve reproducibility and validation across diverse datasets. We argue creating this should be attempted (and is already being attempted) with LLM-based models, given all their advantages. Therefore, once widely-used LLM-based models are created for a given construct, there should be a balance between capturing constructs in slightly better ways with using existing well-validated models, to avoid the same issue of over-proliferation of under-utilized rating-scale assessments [1].

An alternative is building one’s own lexicon, and one can have an LLMs generate a draft lexicon as a starting point [35]. This is likely to achieve improved performance (criterion validity) and higher content validity. Custom lexicons can be used directly without training a model (zero-shot) in the sense that one can count whether their tokens appear in a document without training a model, although it is unlikely to perform very well. It is better to train a model to learn how much to weigh these counts to detect the dependent variable. The main limitations with lexicons is they cannot match tokens that are similar to the ones they have (“suicide attempt” will not match “attempted suicide”) and they lack awareness of how a word’s context might be associated with the target variable (’not sad’,’very sad’,’sort of sad’), which explains their limited performance. Fine-tuning a deep learning model (e.g., BERT) overcomes this issue of context, which is one of the reasons why it tends to outperform lexicons, but requires substantially more training data and are black-boxes [35]. An intermediate approach between lexicons and fine-tuning a deep learning model is to use Construct-Text similarity, which is zero-shot, matches similar tokens, has high content validity, and is interpretable. However, it is also insensitive to context which will limit its predictive performance.

We also note that apparent interpretability of lexicons depends on reporting which tokens drove a construct; because individual matched tokens may not be prototypical of that construct (e.g., “cut” is less prototypical than “BPD” for borderline personality disorder) or may be shared by multiple constructs leading to a biased interpretation of what constructs are most important for prediction. Furthermore, interpretability of a method should always be assessed alongside its predictive performance: the closer a model performs to chance, the less relevant its explanation is for the target application.

Recent LLMs perform well zero-shot, can be fine-tuned for improved predictive performance, and can be requested to provide self-explanations of why they generated a certain result, which may or may not be truthful [73]. They may be sensitive to extremely large prompts (i.e., context windows) and can capture attempts at avoiding keyword detection (e.g., “s3lf harm3d yesterday” would be likely flagged as self harm). Open-source versions currently require substantial computational resources (GPUs, technical abilities) and tend to perform worse than proprietary versions [67]; however, a proprietary version requires users to submit their data to APIs, which may not be permitted for sensitive data. It is also challenging to make generative LLMs output deterministic results, which may create unforeseen errors and is particularly problematic in high-stakes scenarios. Other approaches (BERT-type models) can be used in these settings for deterministic results as an alternative or used alongside generative LLMs.

Overall, LLMs are already dominating other methods given their performance is extremely high on many types of tasks. For supervised text classification with enough labeled data, fine-tuned encoder models (e.g., BERT/RoBERTa) can match zero-shot generative LLMs [110,111]; generative LLMs are most advantageous in zero-shot settings (when limited training data is available or training is undesirable) and likely for many other NLP tasks. LLMs will gain even more traction once their size decrease in order to run more easily on personal computers and their self-explanations are deemed not only plausible, but also faithful (i.e., true) [73]. More mechanistic interpretability methods and understanding for LLMs are also being developed [112,113]. Lexicons or construct-text similarity might be prioritized over other deep learning approaches when (a) an interpretable model is preferred for high-stakes decisions or scientific inquiry (perhaps used in tandem with LLMs to guarantee capturing tokens an LLM might miss); and (b) when models that might result in higher predictive performance are challenging to train or use due to lack of labeled data, computational resources, or data-privacy requirements that forbid sharing data with a LLM API or cost (e.g., running LLMs for millions of electronic health records).

A complementary strategy is triangulation: when a finding replicates across methods with different assumptions (e.g., lexicons, embeddings, language models), confidence increases in the findings (e.g., as in [114], where convergence across three complementary methods strengthened the substantive conclusion); when it appears with one method but not others, it can be treated with caution. Nevertheless, agreement across methods may, depending on the analyses and interpretation, be closer to a form of reliability than to validity: multiple methods may converge yet share the same bias—so triangulation should be paired with the validity checks in Section 4 (e.g., criterion and causal validity). Efforts to share and independently validate language-based models across labs [115] can further support such convergence.

### Are text-based measurements objective if they predict a subjective criterion? Moving beyond the convergence with rating scales

It is commonly thought that a biomarker (digital speech pattern, neural circuit, genetic marker) or behavioral marker (language sentiment) are objective because they can measure variables automatically, that is, they are more objective than any one human’s subjective opinion. However, can these markers be considered objective if they are predicting subjective variables such as a self-report questionnaire? A first consideration is that the target labels may come not from self-report but from the opinion of experts who provided the labels, who may use more objective criteria than nonexperts. The label may be a gold-standard that is relatively objective, such as a diagnosis of vocal-fold paralysis through video endoscopy [116]. A model that accurately predicts expert judgements could be more objective than most non-expert humans. Second, if a model is biased or unfair, such as one that unjustly uses proxies for race to grant or deny parole to incarcerated individuals [117], it could in theory be improved or corrected through bias mitigation methods [96]. This model could end up being more objective than the average judge. Therefore, by increasing the amount of training datasets and correcting over time, a model might become more objective than the humans who provided annotations in any one of its training datasets. Self-supervised models such as generative AI LLMs achieve this even without an external criterion: it uses next-word prediction of the own training data on vast amounts of diverse corpora to allow models to gain rich representations useful in having better predictions than many humans in a variety of downstream tasks, especially when combined with reinforcement learning with human feedback [118]. While bias mitigation methods can reduce systematic errors in theory, it would be premature to conclude that corrected models are more objective than human judges; this claim requires empirical demonstration under controlled conditions, ideally using criterion labels that are themselves verifiably objective.

Nevertheless, given text-based measures often predict subjective target variables, it might be important to evaluate the validity of a text-based measurement beyond assessing its convergence to a subjective rating scale [2]. Several studies have compared text models to rating scales without having to train the text model to optimize the prediction of a self-report rating scale variable. One study [119] took the pictures of facial expressions of different emotions done by actors (happy, sad, or contemptuous, taken as the ground-truth) and randomly assigned participants to either describe each expression with words or rate each stimulus using rating scales for the corresponding expression (e.g., 1 = low to 5 = high happiness). Then both types of responses (i.e., text versus rating scale) were used to predict the ground-truth emotion category and the text-based model showed higher performance. Therefore, these types of analyses allow text-based models to independently be compared to rating scales using criterion validity of a ground truth.

Another study [6] first had one set of individuals write narratives about a series of internal states (e.g., depression, anxiety); these narratives are safely assumed to be about those states (ground-truth). Then, a second set of individuals read these narratives and were asked to evaluate them in two ways: by generating five descriptive words summarizing the text, and by assigning scores using relevant rating scales (PHQ-9, GAD-7). Finally, models were trained to predict the ground-truth states from either the semantic representations of the five descriptive words or the rating-scale scores. The text-based models (using the descriptive words) outperformed the rating scales in accurately detecting the emotional states.

Going forward, –as stated in the Introduction– choosing between natural language and rating scale assessments should weigh their many psychometric properties, assessment time, and whether assessing a given construct or its severity is needed. A person may not mention they are experiencing a symptom or may not clarify how severe a given symptom is, which could be evaluated with a specific rating scale or followup open-text question.

### Ethical considerations of assessing psychological constructs in text

As NLP-based assessments improve, it might be possible for users to assess individuals without their consent. This could lead to spreading private information or rejecting individuals during jobs or insurance applications based on model predictions. European legislation has proposed individuals have a right to an explanation on high-stakes algorithmic decisions, which would make these malicious practices illegal [120]. As models become better, users may not doubt models’ outputs, which is especially problematic if we trust AI to evaluate AI (the section “Other NLP tasks”). If measurement is done via AI chatbots then all the known risks of generative AI hold: hallucinations; suicide encouragement; non-evidence-based medical recommendations; disinformation; illegal, toxic, and discriminatory content; leakage of personal information or copyrighted material from the training set; and plagiarism [121]. Standardized AI safety benchmarks are being used to assess LLMs [122]. Specific questions could be posed to a model to determine whether it will output harmful content such as suicide methods. Furthermore, training, fine-tuning, and particularly running inference with LLMs can produce a substantial impact on the environment, and reducing this impact is an active area of research [123].

### Limitations

LLMs are known to suffer from small changes in the prompts, which can be evaluated more thoroughly (e.g., see [21], who refer to this as a lack of consistency), which is similar to parallel forms validity or intra-method validity in psychometrics [27].

We evaluated different types of models in predicting 13 types of issues. A comparison of these text classification methods (and potentially other methods) on multiple datasets from different domains (e.g., clinical, nonclinical, political) would be useful. While we cannot assume the results will hold exactly in other datasets, we do hypothesize a similar trend in other datasets given the discussed advantages and limitations of each approach (e.g., amount of training data needed, how content validity reflects capturing relevant constructs, the lack of context-sensitivity in some methods, reasoning abilities and general-domain knowledge of newer generative AI models).

To limit the scope of our study, we have focused on comparing text models to rating scale assessments. We have not compared text models to behavioral tasks, qualitative interviews, or other types of data often used in psychology and psychometrics, which would be important to explore in future work.

### Future directions: Exploring other models, other NLP tasks, and AI evaluating AI

In this section, we discuss possible extensions to this type of research, beyond the specific use case of crisis counseling discussed above. These do not reflect any plans or opinions of Crisis Text Line.

#### Other psychometric methods.

In section A of S1 Appendix (“Brief history of psychometrics: from classical test theory to networks and causal inference”), we briefly reviewed factor analysis, item response theory, networks, and causal inference. Other multivariate modeling has been approached in psychometrics using MANOVA (independent regression models) and a series of path analyses such as structural equation models (which integrate confirmatory factor analysis into a path analytic framework), multidimensional scaling [100], and moderator-mediator models [100] that try to provide more insight into associations between variables. Future work could further explore how to combine these methods with text data; for instance, using embeddings instead of rating-scale responses for factor analysis.

#### Other large language model methods.

Multiple methods could be applied to improve performance including few-shot or exemplar prompting (i.e., including multiple examples in the prompt with their correct labels), chain-of-thought prompting (i.e., instructing the model to provide a step-by-step explanation in the prompt before providing the answer), ensemble refinement (e.g., include multiple chain-of-thought responses through temperature sampling in the prompt for the final answer), sampling different hyperparameters (temperature, top-k, and nucleus), and fine-tuning using efficient methods such as QLoRA [70,124].

#### Other NLP tasks.

We focused on text classification, but there are many other NLP tasks that could also benefit from rigorous psychometrics. We briefly outline additional tasks and how they are currently validated. Many of these tasks and metrics can be evaluated before and after deployment.

**NER:** classification metrics can be used as well as more nuanced interpretability methods [125].

**Summarization and question-answering**. Summarization has many applications such as creating artificial memories in generative AI chatbots so it can access information about a user or provide summaries of electronic health records to clinicians before they see a patient. Question-answering also returns a text which needs to be validated to some ground truth. Validation of these tasks involves comparing the similarity to a ground-truth (criterion validity), although recent approach are reference-free [126].

**RAG (Retrieval-Augmented Generation)** is an approach to improve generative AI giving them access to external databases [127]. Even though LLMs are originally trained on large corpora, they will likely not have information that is proprietary or novel and, therefore, can benefit from searching through new data using semantic textual similarity and other techniques. There are many metrics and benchmarks that evaluate different properties of the retrieved information [127]. Applying item-response theory (IRT) could help evaluate whether the system adapts appropriately to varying levels of question difficulty or ambiguity.

**Chatbots**. Evaluating the quality of a chatbot will require similar metrics as we have used, but also ethical considerations as well as metrics related to user engagement and conversation length. Fully automated chatbots likely pose serious risks in high-stakes scenarios such as crisis counseling. A desirable property for a chatbot could be long-term memory of the interactions with the user, which may help the system retrieve details of the user over time and over longer documents. Another may be to have a reliable personality [21].

**Agentic AI**. AI agents can take human input via prompts in order to interact with a device and take actions. Examples include virtual assistants that can set an alarm; AI research assistants that can search for articles, analyze data, and draft manuscripts; and code assistants that can generate and test scripts [128]. They are typically evaluated using task completion metrics, benchmark suites (e.g., BabyAGI, AutoGPT), and qualitative human feedback on their goal achievement and adaptability. Incorporating psychometrics could enhance evaluation by modeling latent behavioral traits and tracking their evolution over time, task type, complexity, and novelty.

**LLM-as-a-judge: AI evaluating AI for automated psychometrics**. If AI is similar or better than humans at a given task, then AI could be the ground truth criterion we try to predict, perhaps to validate smaller, weaker, faster, or cheaper models. This is known as LLM-as-a-judge [129]. The gemma models we used incorporate this during training by having a larger, higher capability model express choose between responses it produces [107]. Models could choose the best hyperparameters of another model for training, suggest adjustments during training, suggest how to adapt trained models to new data, generate synthetic training data to improve measurement, continually monitor predictions once deployed, and run tests to improve predictions. It is feasible that AI could one day help demonstrate all the desirable properties we outlined in the section “Desirable psychometric properties of psychological assessments and how to evaluate these properties in text” on its own. However, generative AI has been shown to carry different types of racial and gender biases [130] as well as methodological biases [129], which should be carefully evaluated and mitigated.

## Conclusion

We have tried to organize and promote research into the validation of NLP-based (including LLM-based) psychological assessments derived from human-generated text. We first presented different types of validity and desirable properties that could be evaluated for text-based assessments. We then systematically compared a diverse set of models including lexicons, zero-shot construct-text similarity, and zero-shot generative AI on different desirable properties in their ability to predict different types of mental health issues and imminent suicide risk. We argue there is a hyperfocus on criterion validity (e.g., out-of-sample prediction of a gold-truth label). We introduced content validity test sets as a way to demonstrate whether models miss any key prototypical expressions of the target variable. This helps us demonstrate that some models can have good criterion validity (e.g., ROC > 0.75) but miss many prototypical tokens (e.g., content validity sensitivity < 0.80). In a second use case, we predicted the need for a future emergency service intervention (i.e., prospective criterion validity) and uncovered which risk factors most predicted the need for an emergency service intervention (e.g., lethal means for suicide more than depressed mood or hopelessness). We finish by providing a comparison of key desirable properties to select models depending on the constraints different researchers may have (Fig 8). As an additional step towards Text Psychometrics, we hope this study will encourage broader validation of these promising NLP methods in psychology, medicine, and the social sciences, and we see much room for future work to extend it.

## Supporting information

S1 Appendix Supplementary methods. A brief history of psychometrics (from classical test theory to networks and causal inference), how to define measurement and validity, a formal description of text classification, a description of construct-text similarity (Fig A), and the prompts used for large language models (Figs B and C).
(PDF)
