# Supplementary material for: Text Psychometrics: Assessing psychological constructs in text using natural language processing
Source: PLOS Digit Health. 2026 Nov 12;5(11):e0001757. doi: 10.1371/journal.pdig.0001757 (PMC13759208; doi:10.1371/journal.pdig.0001757)
Supplement: Supplementary file 1 [file pdig.0001757.s001.pdf]

S1 Appendix

Low, D.M., Mair, P., Nock, M., & Ghosh, S. Text Psychometrics: assessing psychological constructs in text using natural language processing. *PLOS Digital Health.*

A Brief history of psychometrics: from classical test theory to networks and causal inference

Here we review how psychometrics has dealt with modeling psychological assessments throughout its history. From a classical test theory perspective, $X = T + E$ (the true score model) where X is the observed score, T is the true unknown score and E is the unknown error between the two [1]. A good quality measurement should show consistency among repeated attempts to measure the same thing, which implies observations X match true scores T (i.e., reliable scores, free from error across observations). Differences in repeated attempts could be due to measurement error; seasonal effects; the episodic, oscillating nature of the construct; carryover effects (i.e., people remember their answers if the time-lapse is short). Since true scores and errors are unknown, many different attempts at estimating reliability have been developed which involve either correlating multiple test administrations (i.e., parallel forms reliability, test-retest reliability) or calculating reliability across items of a single test administration (internal consistency) [2]. Instead of assuming there is one source of error as in the classical true score model, Cronbach (1972) [3] proposed generalizability theory to account for multiple sources of error (i.e., facets) including items, raters, and measurement instances [1]. A test can reliably return the same score, but be measuring the wrong attribute, that is, not be valid; whereas, a test cannot logically be both valid and unreliable.

Item response theory (IRT, a.k.a., modern test theory) was later developed to account for the fact that different items have different difficulty and discrimination (i.e., how well an item can differentiate between individuals with different levels of the trait) [4]. IRT achieves this by modeling the probability of a person's response to an item as a function of the person's ability and the item characteristics. This can provide more flexible, efficient, and informative testing than classical test theory approaches. When applied to biomarkers or behavioral makers (e.g., allostatic load from blood biomarkers, a psychological construct from natural language), IRT can help identify individual variables that are most informative and least informative for developing a risk assessment score, and determine if individual variables provide information along the entire risk score continuum, or if they provide information for only a portion of the continuum [5].

A limitation of IRT is that it assumes a specific causal structure where observed items (e.g., sadness, anhedonia, fatigue) are caused by an underlying latent variable (e.g., depression), that is, a reflective model [6]. However, as Fried (2017) [7] points out, psychological constructs do not seem to be natural kinds (e.g., gold, water) that are discovered, unchanging, and exist independently of human measurement; rather they are constructed (human-defined), mutable, and have no sharp boundaries. Once constructed, certain psychological constructs seem to co-occur not necessarily because there is an underlying common cause (e.g., depression as a brain disorder causing changes in passive indicators), but because they cause each other (e.g., each symptom having unique brain circuits that affect each other without the need to consider a depression construct). Instead of a natural kind, this can be better thought of a homeostatic property cluster and statistically modeled through networks (i.e., a graph of variables and multivariate associations between them) [7–10] instead of through latent variable models like IRT. Unlike traditional models that view symptoms as passive outcomes of an underlying illness, the network approach sees symptoms as active contributors that causally trigger and reinforce each

other: symptoms do not merely signal the presence of a disorder—they are the disorder [11]. For more on the history of psychometrics, see [12].

A limitation of prior approaches is they do not prove causality as rigorously as structural causal models that use directed acyclic graphs [13] or as the potential outcomes framework [14]. Having said this, the use of directed acyclic graphs is challenging in psychology given acyclicality would rarely hold if constructs are causing each other, but recent advances that drop this assumption have been proposed [15]. Another approach is to use dynamical systems to obtain closed-formed equations or computational models of how constructs interact in a system [16–18]. This approach is promising because, as with networks, it better reflects the fact that constructs (e.g., panic disorder, algebra proficiency) and exposures (cognitive behavioral therapy, racism) are not a single, unitary objects but a system of often nonlinear interacting objects; therefore, applying standard causal inference on the effect of CBT (which is practiced differently by different clinicians and has many active ingredients) on panic disorder may be an ill-posed problem, which complex systems may model better [17]. From this perspective, mental health disorders are systems that are stuck in negative stable states reinforced over time by harmful causal interactions (anxiety ¿ insomnia ¿ fatigue ¿ anxiety).

B How to define measurement and validity?

A common view of measurement in psychology is that of assigning any type of numeral to a construct through some function or rule [19]. This definition broadly applies to many types of relationships. For instance, the degree to which SAT scores is associated with future college success. So what constitutes a valid measurement? A classic definition for validity states that a test is valid if it measures what it purports to measure [20]. Borsboom et al. (2004) [21] argues that a test is valid for measuring an attribute if the attribute exists and if variations in the attribute causally produce variations in the outcomes of the measurement; if they do not, then perhaps one is measuring something different or nothing at all [21]. Markus and Borsboom (2013, p.54) [22] suggest it might therefore be more accurate to think of measurement as assigning quantities related to a real attribute, which should be on at least an interval scale. If the difference between a score at the 10th percentile and a score 1 point above is not the same as the difference between a score at the 90th percentile and a score 1 point above (i.e., it is not interval), then changes in the real attribute assessed would not create precise changes in the score assigned as occurs in more natural attributes such as temperature and well-validated measurement tools such as thermometers. Furthermore, measurement is prototypically asymmetric: a thermometer measures temperature, but temperature does not measure a thermometer's value given the logical causal direction between the two. In this sense, finding that SAT scores correlate with GPA in college is statistically symmetric, so perhaps should not be considered a case of measurement but of prediction, association, or testing. Therefore, Markus and Borsboom (2013) [22] definition of measurement would not apply to most psychological assessments.

C Formal description of text classification

As described in [23], first, a label y for a raw text input x is provided by a human annotator or algorithm. Then a machine learning model p_θ is trained (a function is learned) to map labels and inputs and predict output $\hat{y}$ where θ is the learned parameters. This can be formalized as $\hat{y} = p_\theta(x)$ for a regression setting. In classification settings, we use the maximum a posteriori prediction $\hat{y} = \text{argmax}_y\, p_\theta(y|x)$ where the category y_i with the maximum value is taken among

possible categories y. Measuring psychological constructs in text can be done for binary or multi-class (i.e., multinomial) classification (i.e., categorical output variable) to answer questions such as "Is this text document about construct c?"; through multi-label classification to answer questions such as "Which of these constructs $c \in C$ are present?"; as well as through regression (which in machine learning refers to predicting ordinal or metric output variables) to answer questions such as "Can I detect the severity or degree that construct c is present in a person from a sample of their text?".

D Construct-Text Similarity

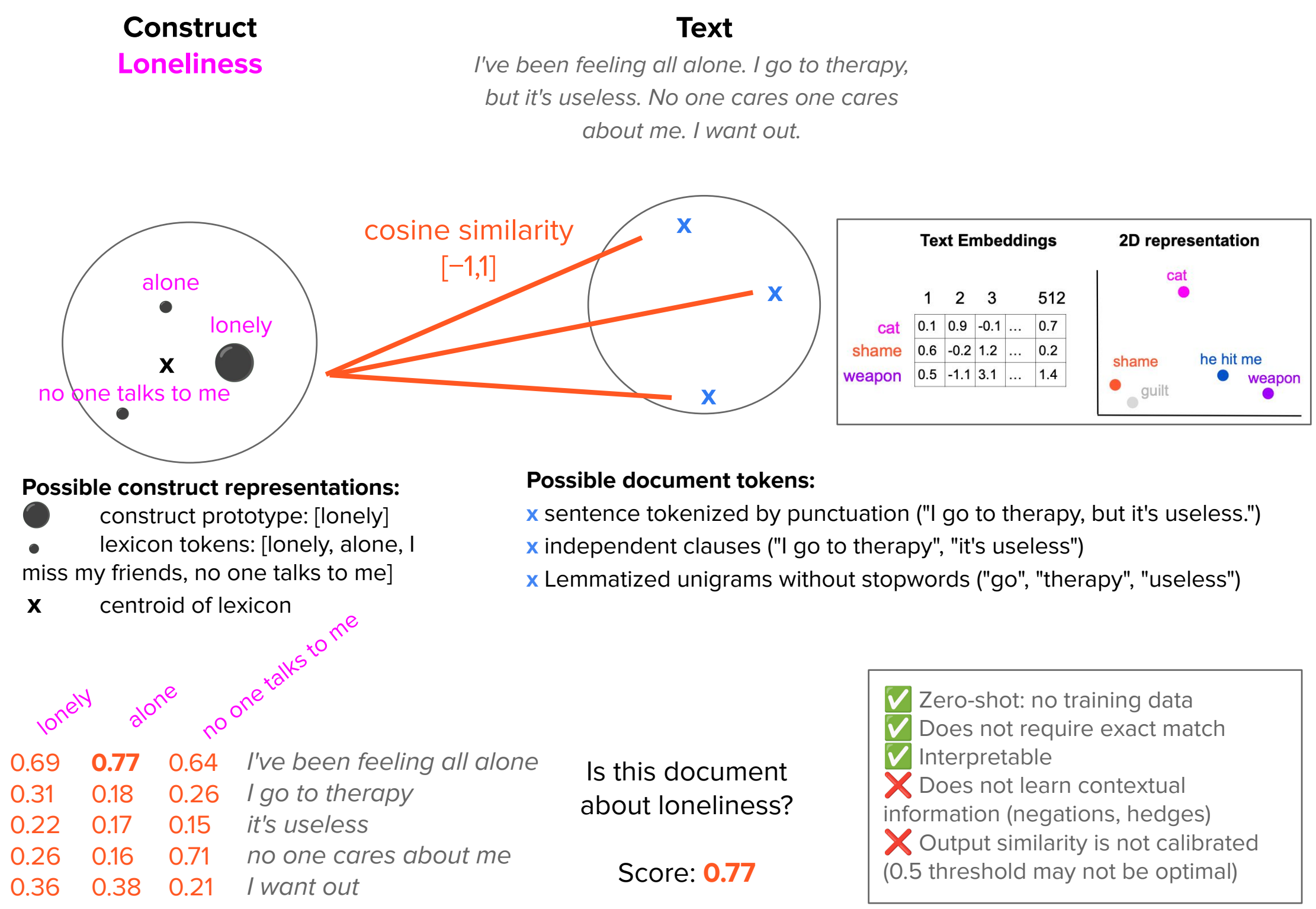

Fig A. Construct-text similarity. Circles and crosses are text embeddings (quantitative representations of meaning in the form of vectors). There are multiple options for computing each step: construct representation, document representation, similarity function, and summary function. Here we build the construct through highly prototypical lexicon tokens, the document representation as independent clauses, compute the cosine similarity, and take the maximum similarity (in bold at the bottom). Conceptually, this is similar to asking where does the document mention something most related to loneliness? The document text is made-up.

E Prompts used for large language models

Small LLMs (e.g., gemma 2b and 7b) performed above chance only with a single classification task (i.e., one issue or label) per prompt (Fig B). The more powerful models (GPT-4o and

Gemini 2.0 flash) could perform well while still including all labels in the same prompt, so as to reduce the number of API submissions.

Prompt

````
You are a <setting> classification assistant. Classify the following <setting> (starts and ends with ```):
```
{document}
```
Assign probabilities for the following labels and return using this JSON format (do not provide additional notes,
explanations, or warnings). Provide your best guess, only return JSON (both probabilities should sum to 1):
JSON:
{'texter mentions something related to anxiety': <your_probability>,
'texter does not mention anything related to anxiety': <your_probability>}
JSON:
````

Output

```
{'texter mentions something related to anxiety': 0.8,
'texter does not mention anything related to anxiety': 0.2}
```

Fig B. Prompt for zero-shot text classification using small LLMs. <setting> could be "Crisis Text Line conversation" or "Reddit post". Prompt was changed for each issue and document. This elicits binary classification.

Prompt

In the following Reddit post, classify the following constructs, defined as follows:

Self harm or self injury: Mention of actual or desired direct and deliberate destruction by an individual of their own body tissue, including with suicidal intent (suicidal self-injury) our without suicidal intent (non-suicidal self-injury). Examples: self-injury; cut myself; cutting; burn myself; harm myself

Anxiety: Anxiety is a feeling of worry, nervousness, or unease, often about an imminent event or something with an uncertain outcome. We include anxiety disorders. Examples: anxiety; fear; afraid; worried; OCD; PTSD; panic attacks; social anxiety; phobia; nightmare; nervous

Suicidal thoughts or suicidal behaviors: Explicit suicidal thoughts of killing oneself; mention of methods, plan, preparations, time, and/or place of suicide attempt. Suicidal thoughts desiring one's own death or related states (e.g., disappearing, not waking up). Suicidal language that does not belong in the other suicidal categories (active and passive SI, self-injury, and lethal means for suicide). Examples: kill myself; jump off a bridge; jump in front of a train; overdose; hang myself; shoot myself wish I wasn't alive; wish I could disappear; hoping I wouldn't wake up; I want to die no reason to live; safety plan; I hate my life

Bullying: Bullying is defined as intentional, repeated, and harmful aggressive behavior (verbal, physical, social) often with an imbalance of power between the perpetrators and the victims. Examples: bullied; makes fun of me

Sexual abuse: Any non-consensual act or behavior of a sexual nature imposed on someone. It can range from unwanted sexual touching and coercion to rape and exploitation. We also include sexual harassment in this lexicon. Examples: rape; sexual assault; was abused; non-consensual; molested me; stalking; groped me

Bereavement or grief: Sadness from losing a loved one (grief) which may result in depressive symptoms (bereavement). This may also include losing a relationship or the health of a loved one. Examples: grief; grieving; bereavement; bereaving; divorce; breakup; died; passed away

Loneliness or social isolation: Loneliness: aversive state experienced when a discrepancy exists between the interpersonal relationships one wishes to have and those that one perceives they currently have. The perception that one's social relationships are not living up to some expectation (Heinrich & Gullone 2006). Isolation: the expression of solitary behavior that results from peer rejection (Rubin & burgess). Related to thwarted belongingness where the person perceives loneliness, isolation, and a failing to meet one's need to belong (Van Orden et al 2010). Examples: lonely; isolated

Depression: Low mood and sadness that does not go away after a few weeks. Examples: depression; sadness; low mood; melancholy

Gender identity: Gender identity refers to an individual's sense of their self as male, female, transgender, non-binary, genderfluid, agender, or something else. Gender identity is distinct from the cultural roles, behaviors, and attributes expected of women and men based on their sex assigned at birth. Sexual identity, also known as sexual orientation, refers to the pattern of romantic or sexual attraction to others. Common sexual identities include: heterosexual, homosexual, bisexual, pansexual, and asexual. Examples: gay; heterosexual; homosexual; lesbian; pansexual; bisexual; asexual; male; female; nonbinary; transgender; intersex; questioning

An eating disorder or body image issues: Eating disorders are mental health conditions characterized by unhealthy and obsessive behaviors related to food, eating, and body image. Common types include anorexia nervosa, bulimia nervosa, and binge-eating disorder. Examples: anorexia; bulimia; binge; purging; eating disorder

Substance use: Mentions of types of abused substances and related behaviors (snort), measures (8 ball), and states (high, buzz) Mentions of types of alcohol beverages and related behaviors (drinking), measures (shot), and states (drunk). Examples: cocaine; heroin; weed; cannabis; meth; amphetamine; snort; 8 ball; pain killer; opioid beer; cocktail; wine; hangover; drunk; buzz

---

Provide a probability score between 0 and 1 for each construct (0=construct is not mentioned, indirectly mentioned, or not clear, 1 = clear and prototypical mention of the construct) as to whether the text clearly mentions the construct and an explanation (words or phrases from the text that are very prototypical expressions of the construct). Only provide a score for these constructs.

Post: {document}

Structure your response in the following JSON format (no extra text):
{'construct_A': [[score], [words, phrases]], 'construct_B': [[score], [words, phrases]], ...}

JSON:

Output

{"Self harm or self injury": [1.0], ["harming a specific part of my body", "wanna harm"], "Anxiety": [0.3], ["don't want her to get mad at me"], ...}

Fig C. Prompt for zero-shot text classification using proprietary LLMs via an API. Prompt was changed for each document. This elicits multi-label classification (scores do not add to 1).

References

1. Mair P, et al. Modern psychometrics with R. vol. 10. Springer; 2018.

2. McNeish D. Thanks coefficient alpha, we'll take it from here. Psychological methods. 2018;23(3):412.

3. Cronbach LJ. The dependability of behavioral measurements. Theory of generalizability for scores and profiles. 1972; p. 1–33.

4. De Ayala RJ. The theory and practice of item response theory. Guilford Publications; 2013.

5. Liu SH, Juster RP, Dams-O'Connor K, Spicer J. Allostatic load scoring using item response theory. Comprehensive Psychoneuroendocrinology. 2021;5:100025.

6. Edwards JR, Bagozzi RP. On the nature and direction of relationships between constructs and measures. Psychological methods. 2000;5(2):155.

7. Fried EI. What are psychological constructs? On the nature and statistical modelling of emotions, intelligence, personality traits and mental disorders. Health psychology review. 2017;11(2):130–134.

8. Borsboom D. Psychometric perspectives on diagnostic systems. Journal of clinical psychology. 2008;64(9):1089–1108.

9. Cramer AO, Waldorp LJ, Van Der Maas HL, Borsboom D. Comorbidity: A network perspective. Behavioral and brain sciences. 2010;33(2-3):137–150.

10. Borsboom D, Deserno MK, Rhemtulla M, Epskamp S, Fried EI, McNally RJ, et al. Network analysis of multivariate data in psychological science. Nature Reviews Methods Primers. 2021;1(1):58.

11. Roefs A, Fried EI, Kindt M, Martijn C, Elzinga B, Evers AW, et al. A new science of mental disorders: Using personalised, transdiagnostic, dynamical systems to understand, model, diagnose and treat psychopathology. Behaviour Research and Therapy. 2022;153:104096.

12. Jones LV, Thissen D. 1 a history and overview of psychometrics. Handbook of statistics. 2006;26:1–27.

13. Pearl J. An introduction to causal inference. The international journal of biostatistics. 2010;6(2).

14. Hernán MA, Robins JM. Causal Inference: What If. Boca Raton: Chapman & Hall/CRC; 2020.

15. Park K, Ryan O, Waldorp L. Discovering Cyclic Causal Models in Psychological Research. 2023;.

16. Robinaugh DJ, Haslbeck J, Waldorp LJ, Kossakowski JJ, Fried EI, Millner AJ, et al. Advancing the network theory of mental disorders: A computational model of panic disorder. Psychological review. 2024;131(6):1482.

17. Ryan O, Haslbeck J, Robinaugh D. Improving Treatments for Mental Disorders using Computational Models. 2023;.

18. Haslbeck J, Ryan O, Robinaugh D, Waldorp L, Borsboom D. Modeling psychopathology: From data models to formal theories. PsyArXiv; 2019.

19. Krantz D, Luce D, Suppes P, Tversky A. Foundations of measurement, Vol. I: Additive and polynomial representations. 1971;.

20. Kelley TL. Interpretation of educational measurements. World Book Company; 1927.

21. Borsboom D, Mellenbergh GJ, Van Heerden J. The concept of validity. Psychological review. 2004;111(4):1061.

22. Markus KA, Borsboom D. Frontiers of test validity theory: Measurement, causation, and meaning. Routledge; 2013.

23. Riezler S, Hagmann M. Validity, reliability, and significance: Empirical methods for NLP and data science. Springer Nature; 2022.
